# Group cognitive space of online rumors in public health emergencies: a theoretical and empirical study

**DOI:** 10.3389/fpubh.2026.1753674

**Published:** 2026-06-12

**Authors:** Chao Shen, Yimeng Zhang, Yuxing Ding

**Affiliations:** School of Management, Nanjing University of Posts and Telecommunications, Nanjing, China

**Keywords:** clustering analysis, cognitive structure, group cognitive space, online rumors, public health emergencies

## Abstract

**Background:**

Internet rumors related to public health emergencies often trigger public panic and result in resource mismatches. During such crises, the public’s cognition is not fixed but continuously undergoes a dynamic process from initial construction to reconstruction, eventually solidifying into a stable “group cognitive space.” Investigating the structure of this space is crucial for understanding heterogeneous public behaviors and enhancing emergency governance.

**Method:**

This study leveraged the LDA topic model and TF-IDF word frequency analysis, in conjunction with domain ontology, to construct a knowledge-dimensional thematic repository, thereby examining the evolutionary patterns of knowledge across distinct phases of public health emergencies. Additionally, the BTM algorithm and K-Means clustering were employed to excavate thematic elements of online rumors, while emotional orientations were dynamically monitored via Baidu AI’s sentiment analysis technology. Guided by Marzano’s classification theory, a cognitive questionnaire was developed for the classification of 440 valid samples; subsequently, the group cognitive space was analyzed through the integration of social cognitive theory and dynamic systems theory.

**Results:**

The group cognitive space exhibits a three-dimensional structure characterized by “knowledge-theme-emotion.” As the event evolves, its knowledge dimension undergoes continuous refinement, the thematic dimension becomes increasingly enriched, and the emotional dimension grows more intricate—tending toward stabilization amid ongoing reconstruction, thereby giving rise to distinct netizen subgroups with heterogeneous cognitive traits. Cluster analysis further identified five such subgroups: rational active communicators, neutral cautious bystanders, highly knowledgeable yet susceptible diffusers, indifferent and detached observers, and contradictory active participants. Furthermore, targeted online rumor management strategies are proposed for each subgroup.

## 1 Introduction

Public health emergencies have become increasingly prevalent in recent years, and the rapid circulation of misinformation and rumors through digital media platforms has created substantial challenges for global health governance (1). Events such as pandemics, emerging infectious diseases, and large-scale outbreaks often take place under high uncertainty, where unverified or misleading information can quickly spread, disrupt evidence-based decision-making, erode public trust, and provoke inappropriate behavioral responses (2). The contemporary information environment—marked by high-velocity transmission, fragmented content, and the anonymity of social media—further intensifies public anxiety and diminishes individuals’ ability to critically assess the credibility of information (3). Against this backdrop, understanding how the public cognitively processes rumor-related information during health emergencies has become an essential issue within public health communication research.

Existing scholarship on health-related misinformation has largely examined several core areas, including the mechanisms underlying rumor propagation, individual information-processing styles, emotional contagion, and risk communication practices (4). Prior studies have demonstrated that uncertainty, emotional arousal, perceived risk, and network structure jointly contribute to the diffusion of rumors (5). Additional research has highlighted the importance of cognitive biases, health literacy, emotional states, and social amplification processes in shaping how people interpret and respond to misleading information (6, 7). Anchored in theoretical frameworks such as the social amplification of risk framework (SARF), social cognitive theory, and information processing models, these studies have collectively advanced our understanding of rumor dynamics in crisis situations (8). Nevertheless, most existing work concentrates either on individual-level cognition or content-level features, which tends to overlook the more complex, multidimensional, and interactive nature of public cognition during public health emergencies.

Despite these contributions, important gaps remain. First, from a theoretical standpoint, limited attention has been given to the systemic and structural dimensions of public cognition. Existing literature rarely conceptualizes cognition as a multilayered and interactive group cognitive space, leaving the configuration and relational dynamics among different public groups underexplored. Second, from a methodological standpoint, prior research often employs single-dimensional indicators or isolated analytical techniques, lacking comprehensive approaches that simultaneously integrate knowledge, thematic attention, and emotional responses. This restricts the capacity to fully capture the complexity of public cognition. Third, from an empirical standpoint, few studies have systematically examined how distinct cognitive groups emerge and evolve across the different phases of a public health emergency. Consequently, the heterogeneity and temporal dynamics of public cognition—critical factors in rumor environments—remain insufficiently understood.

To address this gap, this study proposes the concept of the “Group Cognitive Space”. Crucially, this space is strongly correlated with the specific emergency event. As a crisis unfolds, public cognition continuously reconstructs; only when the event subsides does it solidify into a stable, describable structure. By extracting information across the entire lifecycle of the event, we aim to measure this stabilized structure through a tripartite “knowledge-theme-emotion” framework. This study advances the literature in three key ways:

(1) Theoretically, it proposes and empirically validates the group cognitive space framework, expanding the analytical boundaries of cognition and emotion in crisis communication;
(2) Methodologically, it integrates multidimensional indicators with clustering techniques to systematically identify heterogeneous public groups;
(3) Practically, it offers differentiated communication strategies and governance insights that support targeted rumor intervention and strengthen public health emergency management.

Guided by these objectives, this study addresses the following research questions:

*RQ1*: What structural characteristics define the group cognitive space in the context of online rumors during public health emergencies?

*RQ2*: How can distinct cognitive groups within this space be systematically identified and categorized?

*RQ3*: How does the group cognitive space evolve across different stages of a public health emergency, and what mechanisms contribute to these changes?

The main contributions of this study are threefold. First, it offers significant theoretical explanatory value by integrating cognitive schemas and affective appraisals, successfully deconstructing the internal mechanisms of paradoxical public behaviors. Second, it shifts rumor governance from reactive fact-checking to proactive institutional strategies (e.g., tailored “knowledge vaccines” and emotional-relational governance) with deep ethical considerations. Finally, the proposed framework possesses broad cross-domain applicability, serving as a universal theoretical lens not only for public health emergencies but also for natural disasters and other major social security incidents.

## 2 Related works

### 2.1 Research on online rumors of public health emergencies

Public health emergencies often rapidly generate a sphere of public opinion that stimulates collective emotions. Through further processing by social media and online platforms, vast amounts of uncertain information emerge. Some of this information becomes distorted or amplified and may even transform into online rumors, ultimately leading to deviations in group cognition and societal polarization. In such an information environment, the frequent emergence and dissemination of online rumors are driven not only by public attention to the event itself but also by commercial incentives and media amplification. Existing studies mainly investigate the phenomenon from thematic and emotional perspectives, revealing the multidimensional characteristics and mechanisms of online rumors.

Thematic research on online rumors during public health emergencies primarily reflects the core concerns of the public. Scholars have examined this from three aspects: topic popularity, topic content, and topic relevance. Zhang and Huang (9) proposed an end-to-end multimodal neural network framework for rumor detection in the health domain to classify and characterize rumor-related themes in public health events. From the perspective of social psychological stress, Liu and Qi (10) depicted the interactive infection patterns among various types of rumor initiators under different stress conditions and constructed an interactive infection model across multiple intervention scenarios. An et al. (11), using the LDA topic model to analyze Twitter and Weibo content during the Ebola outbreak, identified evolutionary patterns and cross-platform differences in focus topics, providing empirical support for emergency information management. Guan et al. (12) applied knowledge graphs to develop a risk case knowledge base for emergencies and proposed a topic extraction method combining linear discriminant analysis with association rules to uncover the semantics of negative public opinion topics.

Research on emotional analysis in online public opinion often employs sentiment analysis techniques to classify emotional tendencies in online discourse and examine the characteristics of public sentiment. Medford et al. (13) categorized netizens’ emotions on Twitter during COVID-19 into six types, finding fear to be the most expressed, followed by surprise and anger, with sadness, disgust, and joy being less prevalent. Choi et al. (14) collected media reports and public comments on H1N1 and Ebola, applying sentiment lexicon methods and transfer entropy to analyze reciprocal influences between media and public emotions. Zhang et al. (15) adopted a BERT-BiLSTM fusion model for fine-grained sentiment analysis of Weibo posts during COVID-19, categorizing emotions into seven types and effectively capturing correlations between emotional fluctuations and evolving public opinion topics. Mittal et al. (16) examined correlations between confirmed cases, recoveries, deaths, and emotions expressed on Twitter, finding a significant positive correlation between recovery numbers and positive emotions, whereas no clear relationship emerged for confirmed or death numbers. Hu (17) used the SnowNLP model to calculate sentiment orientation across different evolutionary stages and inducing factors, revealing time-varying emotional dynamics during public health emergencies.

From the perspectives of content and thematic features, research on online rumors in public health emergencies encompasses dissemination and governance. In the early stages of novel public health events, public misunderstanding is common. Delays in official media reporting may accelerate the spread of rumor-related information (18). Beyond official media, the widespread use of social platforms greatly facilitates rumor dissemination. Pengpeng et al. (19) highlighted the role of self-media as “risk sensors” during COVID-19 and noted that imperfect verification mechanisms create fertile ground for rumors. Varshney and Vishwakarma (20) argued that while social platforms allow users to express opinions, they also enable the circulation of false content and controversies. Vosoughi et al. (21) demonstrated that insufficient supervision on social media provides favorable conditions for rumor creation and propagation. Pankow et al. (22) found that rumors and general inquiries during COVID-19 disseminated rapidly through modern social media, greatly increasing the speed and efficiency of information transmission. In terms of governance, Wang et al. (23), drawing on the SIR model and theories of scientific knowledge and external rumor control, developed the G-SCNDR rumor reversal model and proposed governance strategies involving knowledge dissemination, external intervention, and monitoring guidance. Luo et al. (24) integrated neural networks into rumor classification and built a social media rumor identification system to track and restrict rumor dissemination. Overall, current research on online rumors largely focuses on dissemination strategies, influencing factors, and prevention measures, analyzing dissemination characteristics, constructing dissemination models, and proposing rumor-control strategies.

### 2.2 Research on group cognition

Cognition refers to the processes through which individuals or groups perceive, store, interpret, and apply information, including perception, thinking, memory, and decision-making. Cognitive research originated in philosophy, exploring the nature of thought and memory. From the 19th century onward, psychologists studied attention, knowledge encoding, and memory, as well as the influence of social factors on individual cognition, laying the foundation for the multidisciplinary field of cognitive science. Classical definitions of cognition often center on knowing and thinking, implying a form of “offline processing” beyond neural activities that merely correlate with sensory input (25). As research deepened, scholars began to view cognitive processes as dynamic systems involving perception, information processing, experience accumulation, and decision-making. From a socio-cognitive perspective, cognition is not confined to the individual; rather, it is distributed across people, artifacts, and social practices (26). During information dissemination, cognition determines not only how individuals understand information but also how groups form shared understanding and collective responses through social and informational interactions. The formation of group cognition is influenced by factors such as social network structure, member backgrounds, and internal interactions. Network fragmentation can increase cognitive complexity by fostering task conflict, whereas high network density promotes positive cognitive development by reducing relational conflict (27).

The public cognitive process can be viewed as a complex system of encoding, storing, and reconstructing information (28), characterized by contextual dependence and plasticity. Scholars from communication studies and sociology have analyzed cognition as a core component of information reception and processing, shaping the transition from individual to group-level dissemination and the emergence of consensus or action. In public health information interactions, modes of medical knowledge production and dissemination have shifted substantially: ordinary citizens now participate in health knowledge dialog and sharing through social platforms and forums (29). This decentralized mechanism challenges the exclusive authority of experts and enhances group capacity for health information cognition through multi-actor collaboration. Socio-cultural backgrounds further influence the construction of group cognition; citizen science communicators help deconstruct traditional authoritative discourse and establish more egalitarian interactions between communicators and audiences (30). Thus, group cognition depends not only on information content but also on negotiated consensus formed among actors throughout the dissemination process.

Regarding public cognition of online rumors during public health emergencies, scholars have explored the interconnected dimensions of emotion, theme, and cognitive differences. Corinti et al. (31) examined how emotional elements influence rational behavior during public health crises. Wang et al. (32) proposed a multimodal dual-emotion feature model for rumor detection, integrating visual, textual, and social emotions to improve performance across rumor-detection systems. Based on BERT text clustering and lifecycle theory, Liu and Zuo (33) examined topic distribution and evolution in online rumors, revealing fluctuations in public cognition associated with topic change. Focusing on cognitive differences, Zhong et al. (34) constructed a two-layer network model of rumor dissemination incorporating media and individual differences. Shen et al. (35), using Bloom’s taxonomy and combining decision trees with K-Means clustering, explored cognitive differences in information cognition and attitude evolution among netizens. Li et al. (36) confirmed through simulation that knowledge education and intervention strategies improve individuals’ rumor-identification abilities and reduce dissemination peaks.

In summary, existing literature on public cognition and rumor dissemination during health emergencies can be systematically categorized into three core dimensions: knowledge, theme, and emotion. Health literacy and domain-specific knowledge serve as the cognitive foundation for individuals to process crisis information. For instance, recent studies have demonstrated that individuals’ health literacy significantly influences their risk perception, anxiety levels, and ability to filter misinformation during the COVID-19 pandemic (37, 38). The thematic characteristics of rumors act as situational triggers that capture public attention. Topic modeling approaches have revealed that health rumors predominantly cluster around specific themes (such as prevention measures, disease severity, and social events), which distinctively shape public cognitive focus and behavioral responses (39, 40). Emotion functions as the affective catalyst in information dissemination. Sentiment analysis of crisis-related rumors reveals that negative emotions (e.g., fear and anxiety) not only dominate the initial outbreak phase but also significantly accelerate the dissemination of false information (41).

While previous studies provide profound insights into these individual aspects, they are often investigated in isolation. Therefore, synthesizing knowledge, theme, and emotion into a cohesive “group cognitive space” framework is theoretically imperative. This multidimensional approach bridges the gap between static content analysis and dynamic cognitive evaluation, providing a coherent argument for understanding the complex mechanisms of public cognition. Although existing studies provide extensive insights into the generation, dissemination, impact, and debunking of online rumors, significant gaps remain at the cognitive level. First, research rarely begins from a cognitive perspective, let alone conceptualizes rumor dissemination as a systemic cognitive phenomenon. Most studies focus on topic detection, sentiment analysis, or dissemination mechanisms, with limited attention to how online rumors shape group cognition and its dynamic evolution. Moreover, the cognitive roles of individual netizens are often overlooked. Second, cognitive research tends to emphasize cognitive processes and representations, lacking systematic analysis of cognitive structures, particularly public cognitive structures during health emergencies. Existing studies focus more on behavior, rumor content, and individual emotions, but rarely provide structured or macro-level accounts of public cognition. Given the structural nature of cognition, it is necessary to construct a cognitive space model from the perspective of group cognitive structure to analyze its characteristics and offer new insights into rumor governance.

### 2.3 The connotation of group cognitive space

Spatial categories constitute a fundamental component of human cognitive structure, reflecting the universal phenomenon that all objects occupy specific positions in space. In physics, space refers to the three-dimensional expanse of the universe that accommodates matter and energy. In cognitive psychology, however, space is not merely a container of objects but also a carrier of consciousness. Physical space possesses objectivity and universality, forming the basis of our understanding of the world. Cognitive space, by contrast, is a subjective internal construct shaped through sensory processing of the physical world, and is more flexible and variable. Cognitive space involves the processing of object size, shape, and orientation and is influenced by individual experience, cultural background, and cognitive structure, leading to divergent perceptions of the same object.

Group cognitive space is a nascent research concept in the field of social cognition, and the existing limited literature has formed preliminary research consensus on its core connotation: it is the collective cognitive mental construct formed by the interaction of multiple individuals in the social group, and its formation and evolution are closely related to social network structure, group interaction mode and external information stimuli. Momennejad (42) further pointed out that group cognitive space has the characteristics of “distribution and interaction”, and the social network topology directly shapes the structural form of group cognitive space. However, the existing research on group cognitive space has not yet formed a unified definition and dimension division, and the research context is mostly limited to the general social production and life scenario, without targeting the specific scenario of online rumor dissemination in public health emergencies.

During public health emergencies, public cognition evolves dynamically under the influence of multiple factors and adjusts continuously over time. Cognition emerges from the interaction of classical cognitive processes with sensorimotor modalities, the body, and both physical and social environments (43). Through visual perception and cognitive processing, individuals construct mental representations of the world, thereby forming cognitive space. A mapping relationship exists between cognitive and physical space, allowing the external world to be internally represented and expressed through linguistic symbols. Language reflects an individual’s conceptualization of the world based on lived experience rather than directly corresponding to physical reality. Human cognition is also shaped by collective learning and memory. Human networks transmit and share information to synchronize collective memories, knowledge, and beliefs (42). During rumor dissemination, group cognitive space plays a pivotal role. Rumors spread and gain acceptance particularly in cognitive spaces characterized by information asymmetry or high uncertainty. Effective rumor governance requires identifying the cognitive patterns of specific groups and the factors influencing them in order to guide information dissemination, correct misconceptions, and—when necessary—adjust cognitive structures to foster rational development.

### 2.4 Analysis of the spatial dimension of group cognition in public health emergencies

Building upon the aforementioned evolution of collective cognition, a group’s stabilized attitudes, perspectives (44), and viewpoints toward a specific public health emergency constitute its “group cognitive space.” To accurately map and empirically measure this stabilized structure, it must be deconstructed into specific analytical dimensions. Therefore, targeting the context of online rumors, this study formally constructs a multidimensional framework for the group cognitive space, encompassing three core dimensions: the knowledge dimension, the theme dimension, and the affect dimension (specifically: cognitive level regarding policies and science, sensitivity to rumor themes, and emotional attitudes).

The necessity of selecting these specific dimensions is theoretically grounded in the unique evolutionary nature of public health emergencies, supported by Social Cognitive Theory and the Cognitive Appraisal Theory of Emotion. During sudden health crises, the public initially lacks sufficient cognition regarding the novel threat. As the event unfolds, public cognition undergoes a dynamic process from initial construction to continuous reconstruction, eventually solidifying into the aforementioned stable cognitive structure. To comprehensively depict this spatial structure and its internal heterogeneity, we must extract diverse information spanning the entire lifecycle of the event. Within this tripartite framework, an individual’s knowledge serves as the foundational mental schema for decoding uncertain information; the specific theme of the rumor acts as the situational trigger that activates corresponding cognitive frames; and emotion represents the affective appraisal that drives subsequent behavioral responses. These three dimensions are not isolated but interact dynamically, providing an appropriate and robust framework to capture the multidimensional nature of group cognitive space. This space encompasses three dimensions: the knowledge dimension, the theme dimension, and the affect dimension—specifically, “cognitive level regarding policies and science knowledge related to public health emergencies,” “sensitivity to themes of online rumors during public health emergencies,” and “emotional attitudes toward online rumors.”

During public health emergencies, individuals’ knowledge levels directly influence how they acquire, understand, and disseminate information. Schema theory and knowledge revision theory indicate that cognitive structures are continuously adjusted under sustained external stimuli and gradually stabilize. Social cognitive theory further asserts that cognition, environment, and behavior interact to shape information-processing styles (45). Individuals with richer knowledge reserves generally possess stronger critical-thinking abilities, enabling them to reduce reliance on online rumors and update cognitive structures to correct misconceptions (46). Knowledge-level differences not only determine individuals’ capacities to judge information but also affect their tendencies to accept or disseminate rumors. Thus, this study selects the knowledge dimension as a core dimension of group cognitive space.

From a social cognitive perspective, individuals’ responses to information depend on their cognitive frameworks and knowledge systems. Online rumors during public health emergencies span diverse domains such as politics, health, and finance, eliciting varying emotional and cognitive responses. Scientific rumors, for instance, are more easily identified by those with relevant expertise, whereas individuals lacking such knowledge may misinterpret or misjudge information. Cultural background and social values also shape public sensitivity to rumors (47). These individual differences interact dynamically during rumor dissemination, forming a complex network of information processing and cognition. For this reason, this study adopts the theme dimension as the second major dimension to analyze how different rumor themes influence public cognitive space.

Emotion comprises individuals’ physiological and psychological reactions to external stimuli based on their experiences (48). Researchers typically distinguish between basic emotions (e.g., happiness, sadness, fear, anger) and complex emotions (e.g., shame, guilt, pride) (49). Cognitive theories of emotion hold that after receiving information, the brain filters and encodes it, forming cognitive structures that shape decision tendencies and trigger emotional responses, thereby influencing information processing and dissemination. In online environments, individuals often disseminate rumors aligned with their emotional stance due to emotional resonance or group identity, sometimes overriding rational considerations of truth. Strong emotions can also intensify polarization, making individuals more likely to share information consistent with their emotional inclinations, thereby reinforcing existing cognitive patterns and worsening social division. Thus, this study identifies the affect dimension as a crucial component of group cognitive space to analyze emotional responses to online rumors and their influence on public cognition. Crucially, the theoretical explanatory value of this tripartite framework lies in its capacity to deconstruct the “black box” of public information processing during crises. While prior studies have extensively utilized topic modeling or sentiment analysis in isolation to track rumor trajectories, these methods describe what is happening rather than why heterogeneous groups behave differently. Our framework theorizes that group cognition is not a flat aggregation of opinions, but a structured topological space: Knowledge serves as the cognitive anchor (determining the capacity to evaluate information), Theme acts as the situational trigger (activating specific psychological boundaries), and Emotion functions as the affective catalyst (driving the momentum of dissemination). By integrating these three dimensions, the framework provides a theoretical lens to explain the underlying mechanisms of cognitive dissonance—such as why individuals with adequate knowledge might still amplify rumors when specific thematic triggers overwhelm their rational schemas with intense emotions.

**Figure 1:**
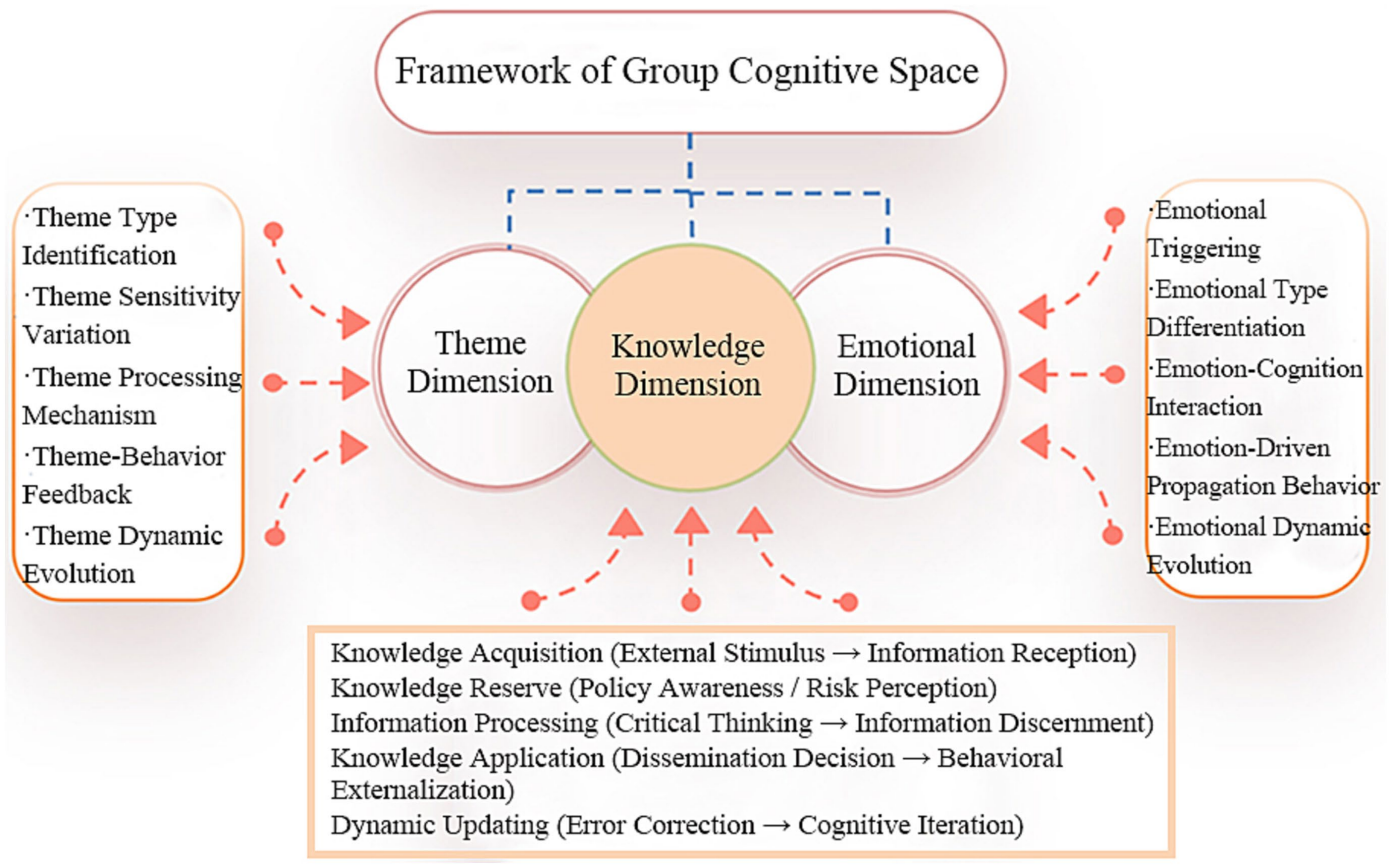
The picture of group cognitive space.

In summary, group cognitive space is shaped by multiple factors, among which the knowledge, theme, and affect dimensions collectively form the public’s cognitive framework for responding to online rumors during public health emergencies (Figure 1). The knowledge dimension determines individuals’ capacity to acquire and process information; the theme dimension influences public sensitivity to different types of rumors; and the affect dimension shapes the emotional context and attitudes toward information dissemination. These dimensions interact dynamically, jointly shaping the evolution of public cognition regarding online rumors during public health emergencies.

## 3 Research design

### 3.1 Research ideas

During public health emergencies, individuals gradually form a relatively stable cognitive framework through iterative cycles of knowledge integration, emotional adaptation, and behavioral decision-making under the dual pressures of information ambiguity and risk uncertainty. This cognitive evolution process provides a theoretical foundation for studying group cognitive space and reveals the dynamic changes in cognitive structures across different stages.

**Figure 2:**
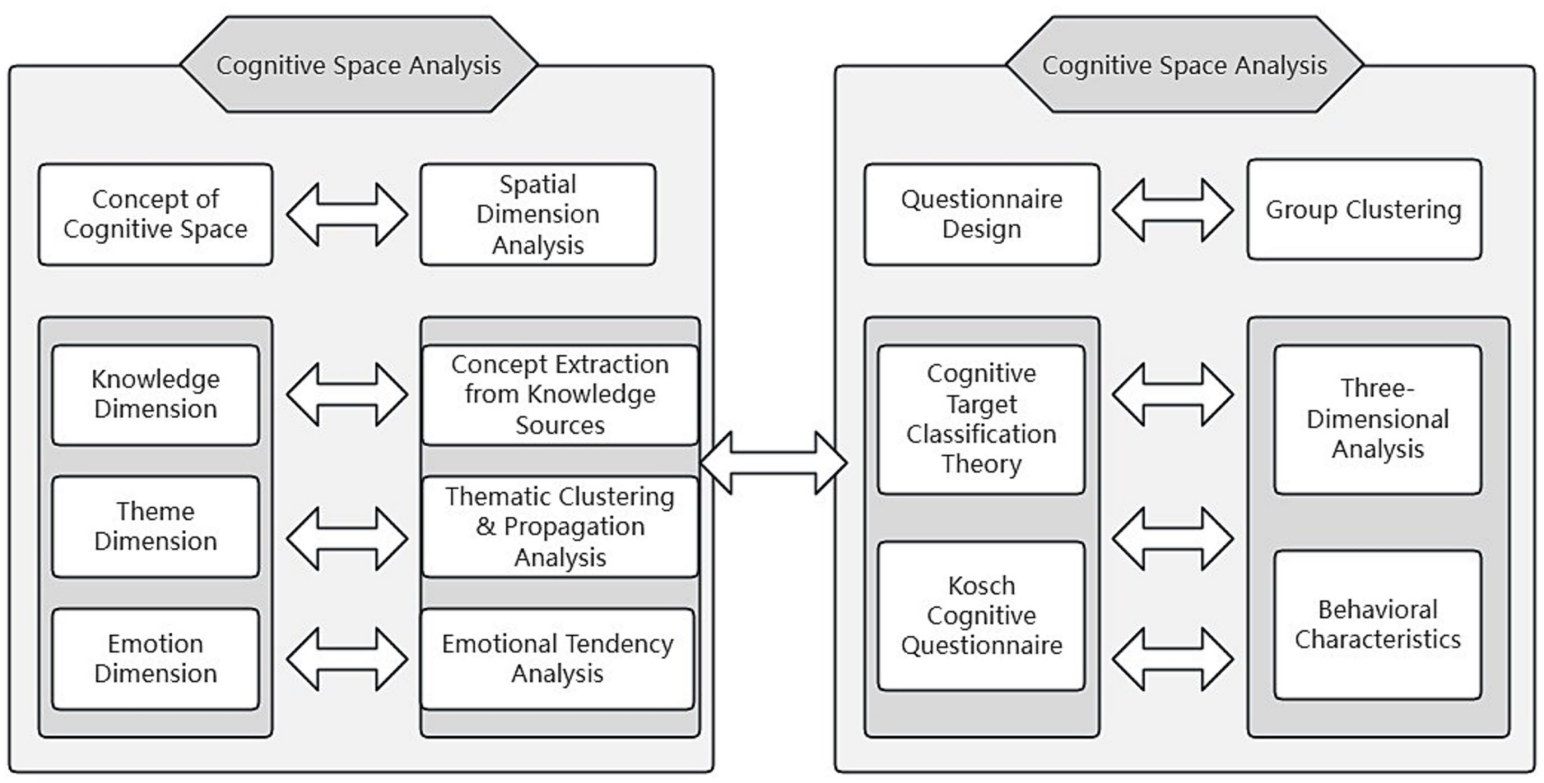
Research framework diagram.

This research is conducted through two primary approaches. First, cognitive space analysis is performed by constructing a group cognitive space model for online rumors during public health emergencies, based on theories of collective cognition. Utilizing techniques including text mining, sentiment analysis, and topic modeling, along with domain ontology, a thematic knowledge base is established to analyze thematic variations and emotional fluctuations of online rumors at different stages. This helps identify public cognitive differences across knowledge, emotional, and thematic dimensions. The objective of this phase is to understand the mechanisms of online rumor dissemination and provide theoretical support for rumor governance. Second, based on the three-dimensional structure of group cognitive space—knowledge, theme, and emotion—public cognitive research questionnaires are designed and distributed. Group classification analysis is then conducted integrating social cognitive theory and dynamic systems theory. Through cluster analysis, characteristics and behavioral patterns of different groups within the cognitive space are identified, offering new perspectives and methods for targeted governance of online rumors (Figure 2).

### 3.2 Deconstruction of the cognitive space of online rumors in public health emergencies

#### 3.2.1 Research on the knowledge dimension of cognitive space

The knowledge dimension is the core cognitive basis for the public to identify online rumors. During public health emergencies, netizens often lack a systematic knowledge framework for understanding emerging risks. Governments therefore disseminate scientific explanations and prevention guidelines through official science communication and policy documents so that the public can better participate in emergency response. These materials provide the knowledge foundation for constructing the public’s cognitive space regarding the event and influence how individuals evaluate rumor-related information. The official knowledge system contained in the corpus forms the basic cognitive framework of netizens, enabling them to compare the content of online rumors with authoritative scientific knowledge and policy information and thus develop the ability to judge rumor credibility. For example, many rumors concern disease transmission routes or unverified treatment methods. If authoritative explanations of such issues already exist in the knowledge corpus, users can compare rumor claims with verified information and are more likely to reject misleading content. This cognitive comparison process reflects the mechanism through which the knowledge dimension operates within the group cognitive space of online rumors and provides a basis for analyzing how netizens’ knowledge reserves influence their rumor cognition and behavioral responses.

The document information of public health emergencies is diverse, covering a wide range of fields and numerous concepts. This article conducts a case study by referring to professional literature in related fields and reusing existing ontologies in related fields, taking the COVID-19 pandemic as an example. Referring to the “COVID-19 Prevention and Control Plan (10th Edition)”, the core concepts of the field are determined based on nine parts: etiological and epidemiological characteristics, vaccination, personal protection and publicity and education, monitoring and early warning, testing strategies, prevention and control of key links, emergency prevention and control measures during the epidemic period, and organizational guarantee. See Table 1 for details.

**Table 1:** Core concepts in the field (partial).

Knowledge type	Core concept	Core concept
Etiological and epidemiological characteristics	Etiological and epidemiological characteristics	The main research focuses on the pathogen characteristics, transmission routes, susceptible populations and epidemic patterns of diseases.
Personal protection and publicity and education	Personal protection	Educate the public on how to wear masks correctly, wash hands frequently, maintain social distancing, etc., to reduce the risk of infection.
	Publicity and education	Popularize prevention and control knowledge through various channels to enhance the public’s awareness and ability of prevention and control.
	Patriotic health campaign	Advocate a healthy lifestyle, improve environmental hygiene, and reduce the risk of disease transmission.
Vaccine matters	Vaccination	Ensure the safety and efficacy of vaccines, while strengthening the management of vaccine supply and vaccination sites.

Based on the collected data as the “COVID-19 pandemic” corpus, we crawled policy documents and popular science texts released by official authoritative platforms such as the National Health Commission of China and the Chinese Center for Disease Control and Prevention during the pandemic. By querying the ontological concepts in the fields of medicine, healthcare, logistics, etc., and communicating with 6 doctors and government officials the relevant domain terms were manually identified and combined with the new word discovery function of the NLPIR software to collect them. These terms were then imported as user-defined dictionaries to improve the accuracy of the vocabulary. The domain term collection was integrated with online rumors and policy popular science texts. It constitutes a word segmentation dictionary in the field of “COVID-19” online rumors, as detailed in Table 2.

**Table 2:** Partial dictionary of word segmentation in the field of “COVID-19” online rumors.

Vocabulary	Weight	Vocabulary	Weight
Medical institution	166.32	Resumption of classes	99.93
Confirmed case	161.49	Medical sewage	98.23
Public legal services	141.19	Antiviral	96.81
Resumption of production and work	139.08	Body temperature measurement	93.39
Cold chain logistics	136.12	Caring service	91.18
Older adult care services	127.66	Joint prevention and control	90.01
Medical mask	127.44	Study power	89.65
Drug regulatory department	124.93	Body temperature measurement	88.97
Emergency duty	110.38	Cleaning and disinfection	88.93
Safe childbirth	109.98	Construction	86.06

**Figure 3:**
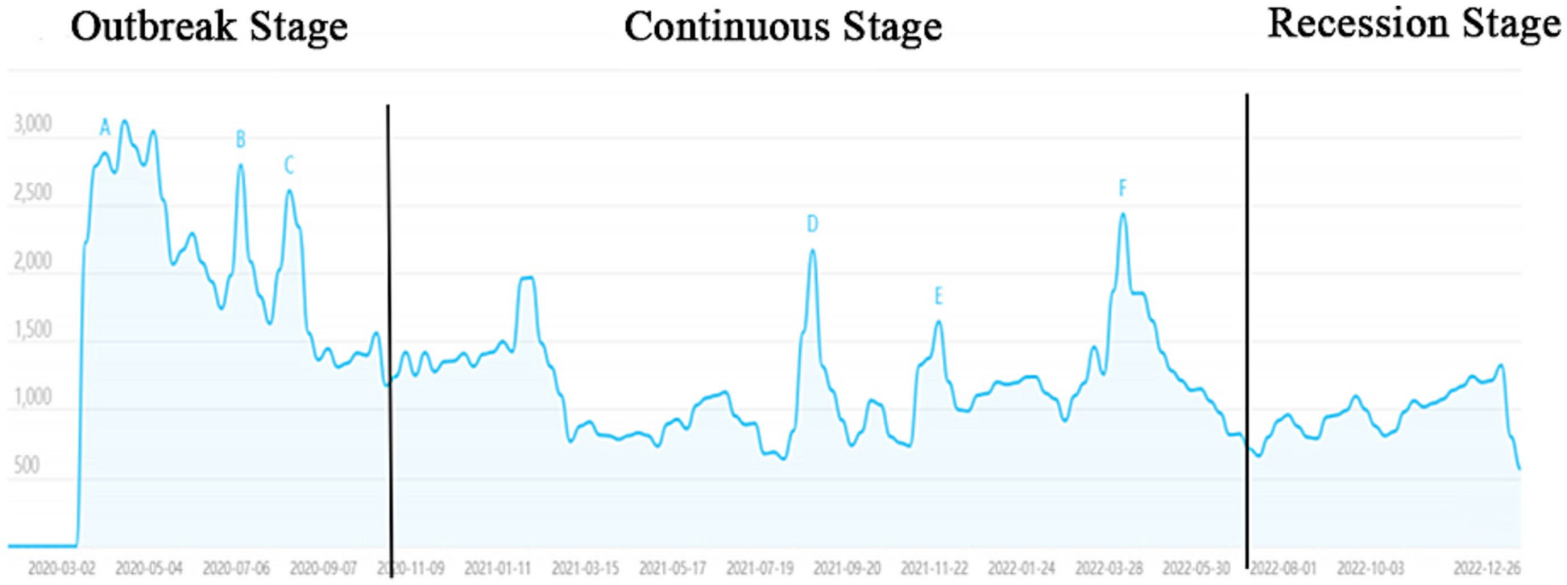
Baidu hot search index trend.

Based on Fink’s life cycle theory (50) and the trend of Baidu Index over time, as shown in Figure 3, the life cycle of online rumors is divided into three stages: the outbreak stage, the persistence stage, and the decline stage (4). In the outbreak stage (early 2020), the core keywords focused on “work—prevention and control—epidemic”, corresponding to the large-scale outbreak of the original COVID-19 strain in Wuhan and the national launch of the first-level emergency response mechanism, specifically manifested in the declaration of a state of emergency, the allocation of medical resources, and the formulation of emergency plans. In the persistence stage (late 2020–2022), the keywords shifted to “personnel—protection—isolation”, corresponding to the repeated local epidemics caused by the Delta variant in Shijiazhuang, Nanjing and other cities, the refinement of national prevention and control guidelines, the dynamic disclosure of epidemic data, and the construction of a mental health service system. In the decline stage (2022), the cognitive focus evolved to “vaccine—economy—recovery”, reflecting the prevalence of the Omicron variant, the optimization of national epidemic prevention and control policies, and the implementation of large-scale vaccination and economic relief policies.

Using the term frequency analysis technology of the TF-IDF algorithm, weighted data was generated to obtain the key words with greater influence in each stage. The larger the value, the higher the importance of the word and the more representative it is. After calculation, the top 10 entries (Table 3) were selected.

**Table 3:** Values of key terms in the three stages (partial).

Outbreak stage		Continuous stage		Recession stage	
Entry	Numerical value	Entry	Numerical value	Entry	Numerical value
Work	0.0139	Epidemic	0.01486	Epidemic	0.01438
Prevention and control	0.01275	Prevention and control	0.01375	Prevention and control	0.01358
Epidemic	0.01205	Personnel	0.01353	Work	0.01172
Personnel	0.01022	Street	0.0126	Enterprise	0.01012
Do a good job	0.00854	Nucleic acid testing	0.01182	Service	0.00748
Implementation	0.00823	Work	0.00908	Personnel	0.00724
Strengthen	0.00804	Community	0.00878	Do a good job	0.00678
Health	0.00733	Isolation	0.00807	Strengthen	0.00615
Disinfection	0.00706	Residential area	0.00796	Pneumonia	0.0061
Requirements	0.00692	Sampling	0.00764	Implementation	0.00517

**Figure 4:**
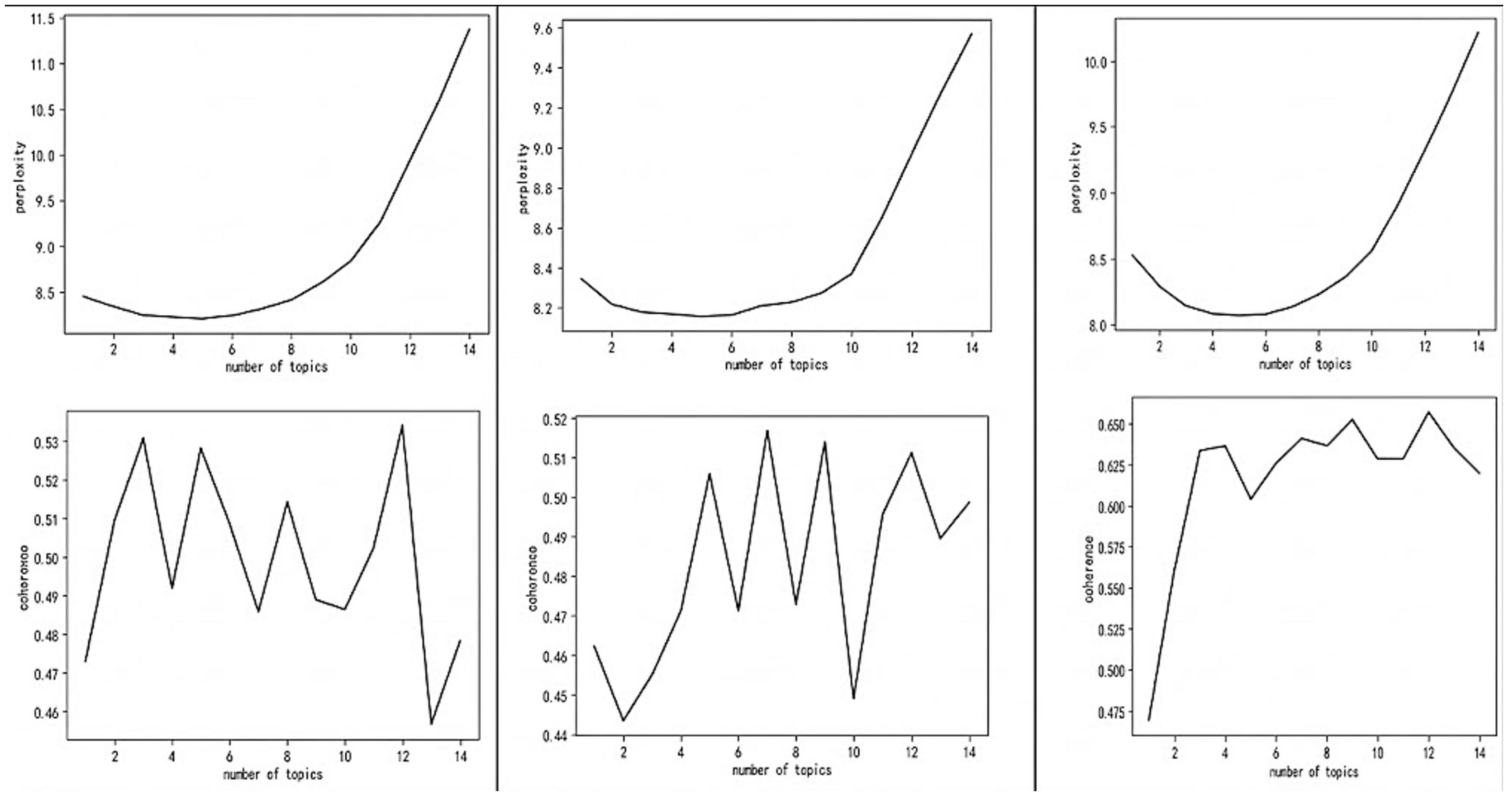
Confusion and consistency curves (from left to right, each column represents the confusion and consistency curves of the outbreak stage, the persistence stage, and the decline stage in sequence. The vertical axis of the first row of the chart is the confusion, the vertical axis of the second row is the consistency curve, and the horizontal axis is the number of themes).

The study employed the LDA topic model to analyze the structure of the knowledge space and utilized the Gensim library to achieve vectorized representation of the text. Based on the dual-index evaluation of perplexity and consistency score, the optimal number of topics for each stage was determined: 5 topics for the outbreak period, 7 topics for the continuation period, and 7 topics for the decline period. As shown in Figure 4, the perplexity curves of the three stages first rose and then fell, while the consistency scores maintained a steady growth, indicating that the group’s cognition gradually evolved from fragmentation in the early stage to structuring.

Through the analysis and research of the extraction results of the themes, it is found that in the outbreak stage, continuous stage and decline stage of public health emergencies, the evolution of knowledge themes shows the characteristics of continuity and distinctiveness. Among them, “epidemic prevention and control measures” and “personnel protection” have been continuously monitored throughout the entire event cycle, reflecting the public’s high concern and demand for health and safety. In the initial stage, the “cultural promotion work” had a significant impact, indicating that the public was prone to panic due to insufficient information, and it was necessary to enhance confidence in epidemic prevention and control measures. At different stages, the attention paid to other themes also varies. For instance, in the explosive stage, the focus is on “cooperation between enterprises and localities”; in the continuous stage, it is on “matters related to going out”; and in the final stage, it is on “promoting economic recovery”. The epidemic prevention and control situations in various regions are also reflected at different stages. For instance, during the outbreak stage, there is the “national overall deployment of epidemic prevention and control work”; during the continuous stage, there is the “local epidemic prevention and control situation”; and during the recession stage, there is the “domestic epidemic prevention and control situation” and the “epidemic prevention and control situation in border port areas”. Especially in the mid-term stage, the attention paid to “vaccination matters” and “building an immune barrier for the population”, as well as in the final stage of “receiving the third dose of the COVID-19 vaccine”, demonstrate the public’s high level of concern for knowledge related to vaccination. It requires multi-party cooperation to arrange and promote the vaccination work, including the collaboration of multiple entities such as the government, schools, and communities.

#### 3.2.2 Research on the thematic dimension of cognitive space

Public health emergencies have led to a large number of online rumors with diverse themes, which makes there both commonalities and differences among the rumors. Based on the three stages divided in the previous text, this paper selects Chinese Internet rumor-refuting platforms to obtain rumor data. Rumor Filter and Tencent Jiaozhen and other platforms are used as supplements. A total of 2,435 rumors from January 1, 2020 to December 31, 2022 are selected. Jieba is used for text word segmentation and combined with the stop word list of Harbin Institute of Technology to clean the segmented content. A total of 2,101 valid data pieces were obtained.

**Figure 5:**
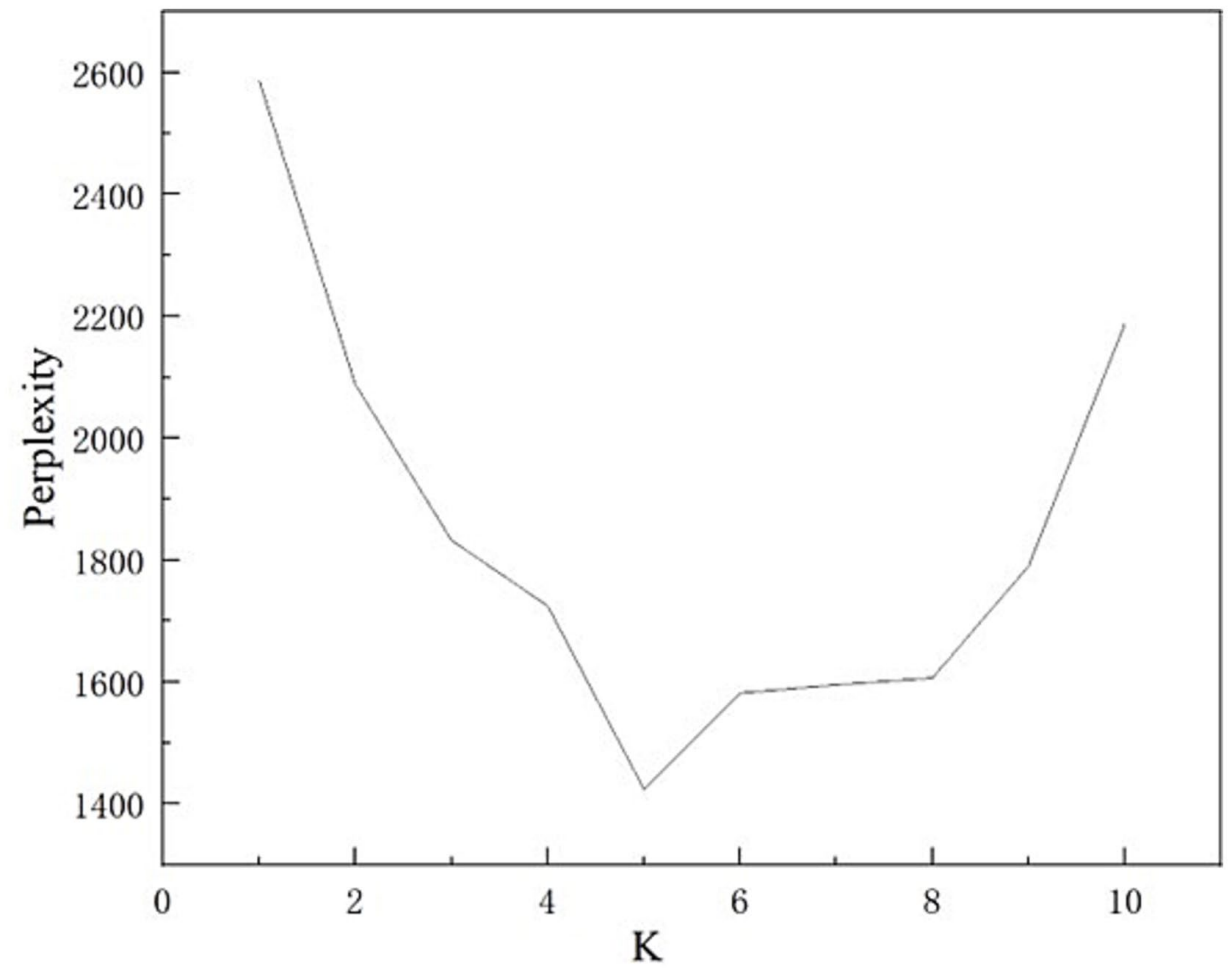
Trend of confusion degree changes.

Based on the BTM topic modeling for K-Means clustering, the optimal number of topics was determined to be five (Figure 5). The clustering results were classified as prevention and control measures, epidemic panic, daily life, virus spread, and social events. The specific feature words are shown in Table 4.

**Table 4:** Extraction of key words for online rumors in public health emergencies.

Topic	Topic words
#1	Nucleic acid, testing, prevention and control, measures, notice, confirmed case, isolation, staff, COVID-19, pneumonia, health, negative, platform
#2	Information, rumors, debunking, videos, the Internet, messages, netizens, epidemic situation, verification, dissemination, reporting center, reporting
#3	Journalist, News, wechat, Supermarket, China, Beijing, Enterprise, Import, Rumor Debunking, Test Kit, Fluorescence, Department, Prevention and Control
#4	Vaccines, viruses, epidemics, infections, pneumonia, novel coronavirus, masks, cases, treatments, tests, populations, discoveries, reports
#5	Sampling, Zhong Nanshan, Academician, Antigen testing, sample, testing, tap water, Internet, news, Hubei, message

To gain a more comprehensive understanding of the characteristics of each topic, select the top 10 feature words. The first category of themes is closely related to the epidemic, revolving around terms such as “people” and “patients,” and involving characteristic words of regional epidemic prevention and control, such as “lockdown” and “testing,” etc. The second category of themes mainly involves feature words related to information dissemination. The third category of themes, in light of the development trend of the epidemic, has seen the emergence of rumors related to events such as students’ reopening and social security. The feature words of the fourth category of themes involve virus prevention and health care treatment. The fifth category of themes covers rumors about some public figures and epidemic events. The themes of online rumors about public health emergencies are shown in Table 5.

**Table 5:** Classification of online rumors related to public health emergencies.

Category	Meaning
Protective measures type	Online rumors related to epidemic prevention and control measures mainly focus on false statements and misleading information about epidemic prevention and control measures. These rumors include incorrect personal protection methods, false promotion of specific foods or medicines, or discriminatory remarks about certain regions or groups of people, etc., which lead the public to misunderstand epidemic prevention and control measures and thereby affect the public’s awareness and behavior of protection.
Panic-creating type	Online rumors of epidemic panic often spread false information about the location of the epidemic outbreak, exaggerate the severity of the epidemic, or disseminate false epidemic data, etc., which have a negative impact on the public’s mood and confidence.
Production and living type	Rumors about the abnormal operation of regional institutions such as public welfare institutions and living places, as well as the looting of epidemic prevention and living supplies and financial fraud, have been widely spread, causing confusion and unease among the public and also leading to the obstruction of some living needs
Virus spread type	Spreading false information about the channels of virus infection, treatment drugs and the infection situation of the epidemic, exaggerating the severity of the epidemic or misleading the public about the spread of the virus.
Social figures type	Online rumors related to public figures and social news events

Panic rumors about the epidemic spread due to the public’s panic mentality. The public is worried about their health, afraid of the unknown, eager for epidemic information and prone to accepting rumors. Some individuals or organizations use social media and the Internet to spread false information for specific purposes, exacerbating panic. Rumors about prevention and control measures spread easily due to the untimely and opaque disclosure of government information and the difficulty in collecting information at the beginning of the epidemic, which led to delays in their release. The public is eager for epidemic information but suspicious of government information. Rumors related to virus spread and daily life, such as exaggerating the harm of the virus and false information about food and beverages, have caused public panic. Although social event-related rumors receive high attention, they are often quickly confirmed or refuted. In response to these rumors, the government, the media and relevant individuals should provide accurate information to prevent the spread of panic.

#### 3.2.3 Research on the emotional dimension of cognitive space

**Figure 6:**
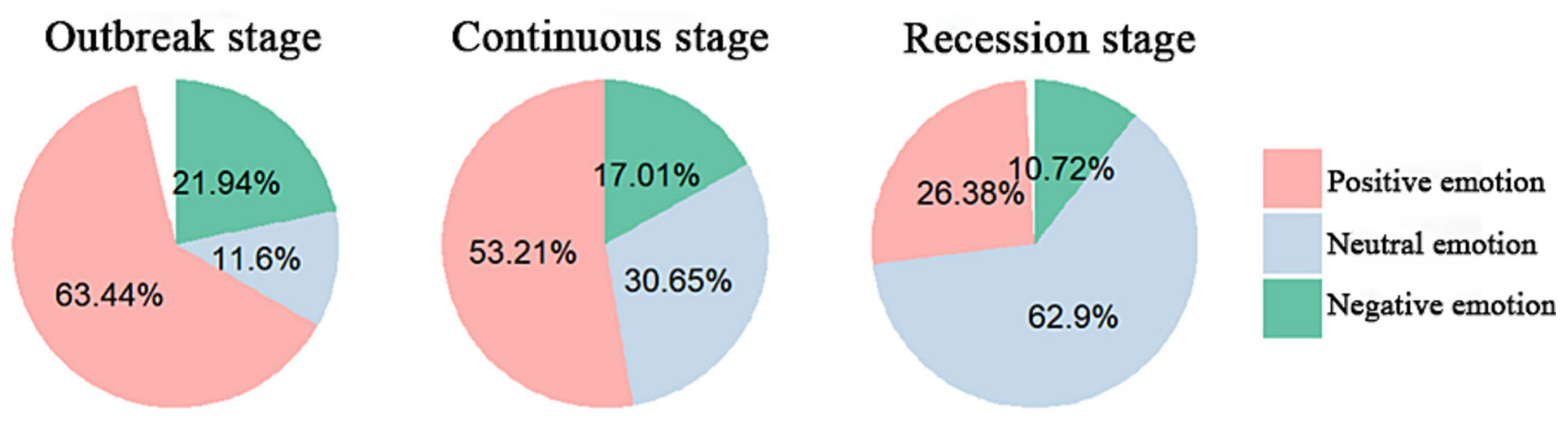
Distribution of three-stage emotional tendencies in online rumors of public health emergencies.

In order to systematically analyze the text presentation trend and emotional tendency of rumors about public health emergencies, by leveraging the emotional analysis function provided by the natural language processing module under Baidu AI Open Platform, This platform employs a deep learning model based on BERT, which is trained on over 10 million Chinese texts with sentiment labels. According to reports, its accuracy in three types of sentiment classification is relatively high, and this result has been verified in peer-reviewed research. Hou et al.’s (51) research utilized the API sentiment analysis interface developed by Baidu platform for sentiment analysis. By calculating the sentiment scores, the researchers were able to comprehensively quantify the emotional fluctuations of the public during different stages of the epidemic. Under Baidu AI Open Platform, the emotional tendency analysis of the obtained rumor data is conducted. The frequency of neutral, positive and negative words is extracted through confidence probability, thereby determining the emotional tendency of each text. The emotional tendency frequency of all online rumor texts related to public health emergencies (Table 6), and the emotional distribution at different stages is shown in Figure 6.

**Table 6:** Statistics on emotional tendencies of online rumors in public health emergencies.

Type	Frequency	Proportion (%)
Positive emotion	592	28.18
Neutral emotion	162	7.71
Negative emotions	1,347	64.11
Total	2,101	100

**Figure 7:**
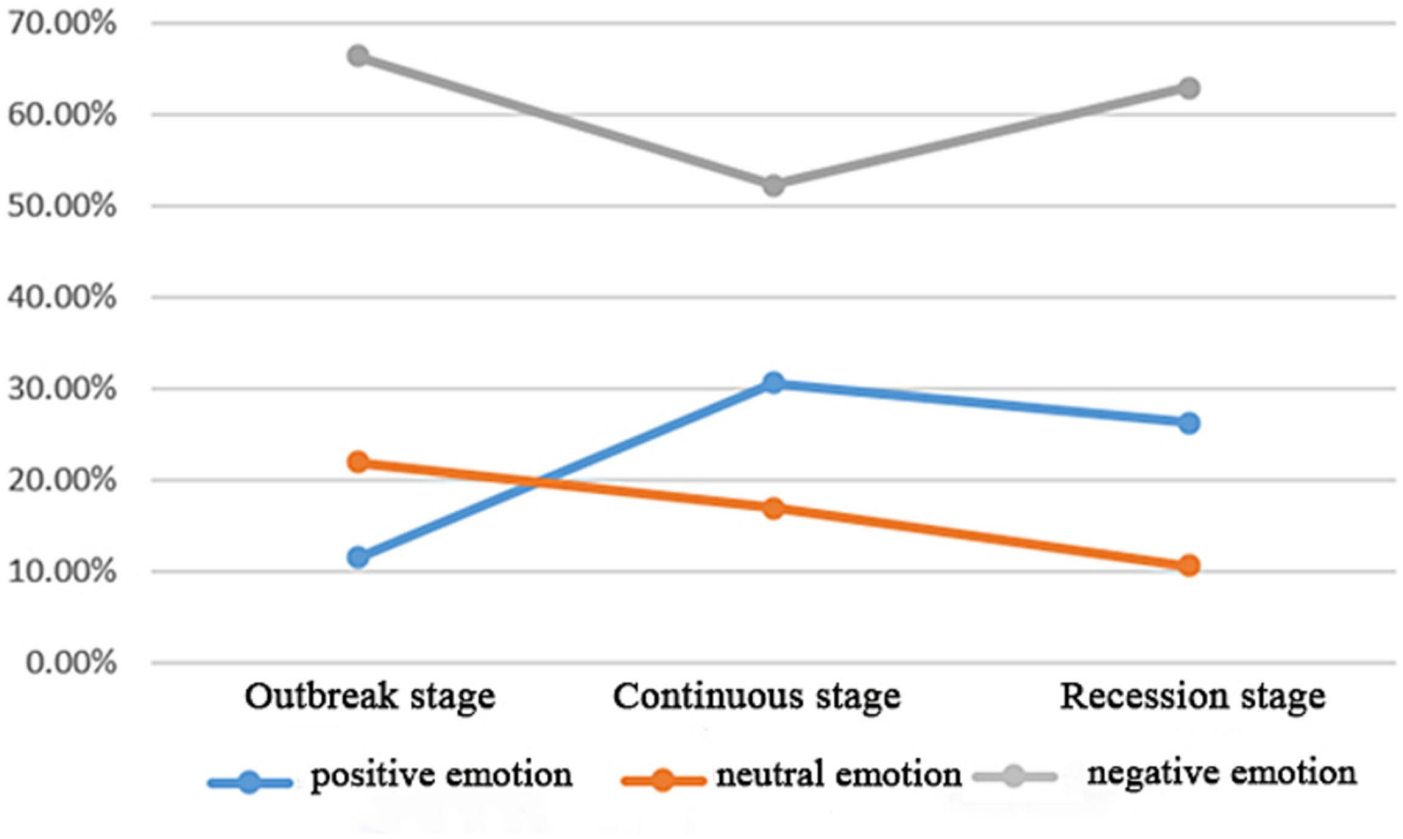
Emotional evolution of online rumors in public health emergencies.

Fitting the rumors and emotional trends, the emotional evolution is consistent with the outbreak of the epidemic, and the overall negative emotions are at a high level. Analyze the changes in rumor emotions along with the epidemic from the perspective of time evolution and draw an emotion evolution graph (Figure 7).

During the outbreak of the epidemic, people often felt panicked and uneasy about the new disease, and this emotion was usually manifested in online rumors. At this stage, rumors with negative emotional tendencies dominate the market, spreading terrifying and pessimistic information and triggering public panic and anxiety. As the epidemic develops and prevention and control measures are implemented, after entering the continuous stage, rumors with negative and positive emotional tendencies may tend to balance. On the one hand, although negative emotions still exist, they have weakened compared to the early stage. People have gained a deeper understanding of the epidemic and are treating information more rationally. On the other hand, rumors with positive emotional tendencies have also begun to increase, spreading hopeful and inspiring messages to alleviate public anxiety.

However, during the recession of the epidemic, rumors with negative emotional tendencies tend to increase significantly. This stage may be accompanied by local outbreaks, an increase in cases and social instability, leading to a resurgence of public panic and anxiety. These emotions are also reflected in online rumors. Relatively speaking, rumors with positive emotional tendencies may show a fluctuating downward trend, but they still exist. According to the analysis, the proportions of negative, positive and neutral rumors are 64.11, 28.18 and 7.71%, respectively. Among them, neutral rumors are mainly concentrated in social event-related and daily life-related rumors.

### 3.3 Questionnaire design

**Figure 8:**
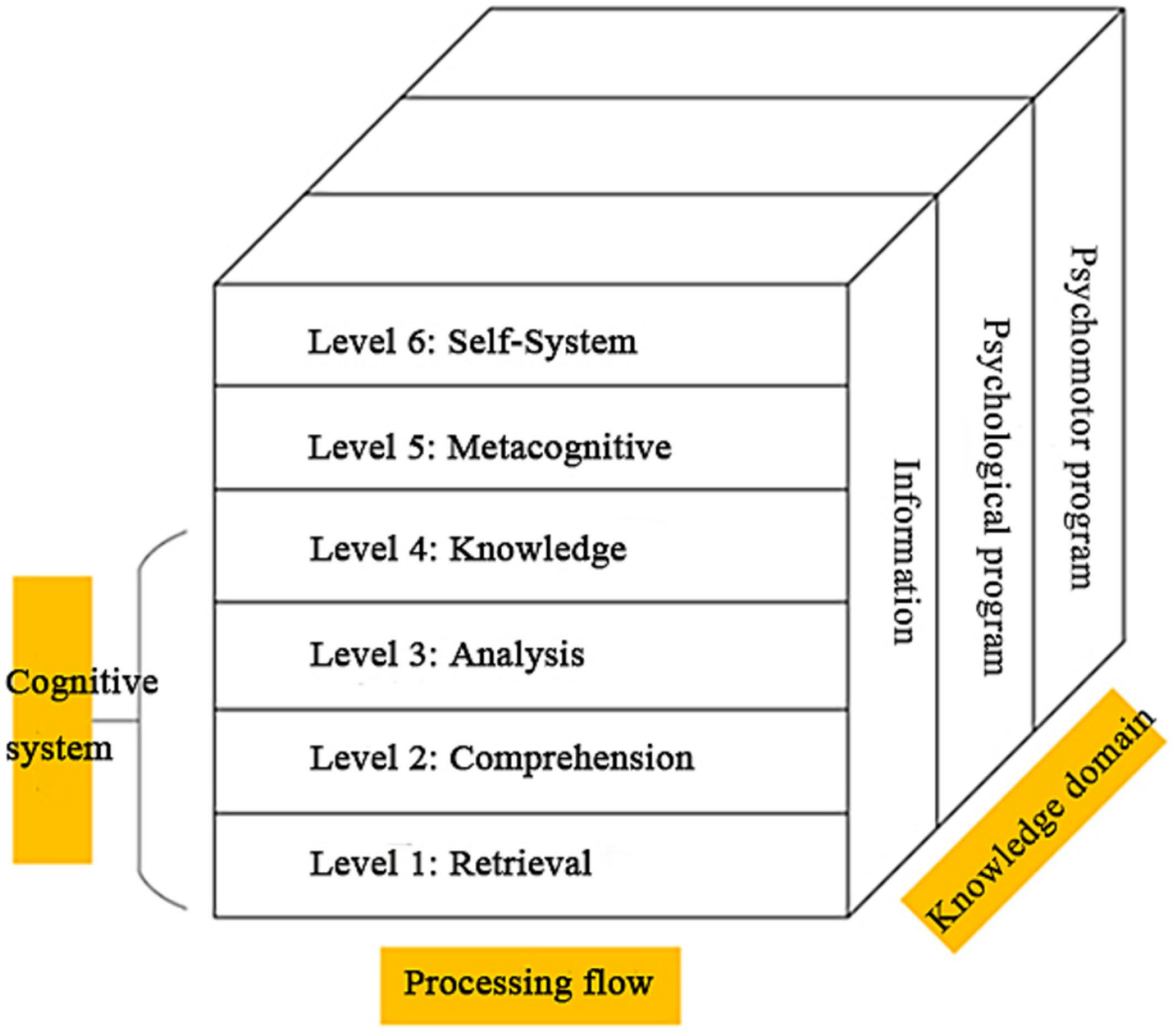
Diagram of the Mazano classification model.

When confronted with uncertain information such as rumors, the various stages that netizens go through in the cognitive process will trigger diverse response behaviors, and these reactions are influenced by multiple factors such as individual knowledge level, judgment ability and emotional attitude. To empirically measure this complex cognitive space, we designed a public perception questionnaire (provided in full as Supplementary material 1). The design was strictly grounded in Marzano’s New Taxonomy of Educational Objectives (52). As illustrated in Figure 8, this taxonomy comprises a two-dimensional framework: the Knowledge Domain and the Systems of Thinking (which includes six levels of cognitive processing: retrieval, comprehension, analysis, knowledge utilization, metacognitive system, and self-system). Our questionnaire items directly map onto this theoretical framework: (1) The Knowledge Domain was assessed through 29 items evaluating respondents’ mastery of 9 core epidemiological concepts (extracted via domain ontology and the LDA model); (2) The Self-System (which dictates motivation and affect) was operationalized through 10 emotional and behavioral response items; (3) The Cognitive System (involving comprehension and analysis) was tested by presenting respondents with 5 typical rumor cases to assess their discernment capabilities.

It is important to clarify the logical relationship between the three temporal evolutionary stages (discussed in Sections 3.1 and 3.2) and the thematic design of the questionnaire. While the three stages describe the macro-level dynamic evolution of rumor topics over the lifespan of the emergency, the questionnaire (conducted in April 2023) was designed to measure the public’s relatively stabilized cognitive structure after the event. Therefore, the five typical rumor themes presented in the survey (i.e., protective measures, panic-creating, production/living, virus spread, and social figures) are not isolated to any single temporal stage. Instead, they represent the cross-sectional, archetypal thematic categories distilled via the BTM algorithm from the entire evolutionary process. This design effectively bridges the macro-level temporal evolution of rumors with the micro-level structural assessment of group cognition. During the design and revision of the questionnaire, Mazano’s cognitive objective classification theory and the Kosch cognitive survey questionnaire (53) were referred to, and the rationality and accuracy of the questionnaire item Settings were verified through two rounds of pre-tests.

Since this study specifically focuses on the dissemination and cognitive processing of online rumors, the target population for the empirical survey is explicitly defined as “active netizens” (predominantly younger, educated individuals who frequently engage with digital information). Demographics with limited internet behavior or lower digital literacy, such as children and older adults, fall outside the scope of this specific empirical investigation.

Prior to formal distribution, two rounds of pre-testing were meticulously conducted to verify the rationality and accuracy of the questionnaire items. To capture the public’s stable cognition following the peak of the pandemic and mass vaccination, the formal survey was conducted from April 1 to April 30, 2023. Regarding the temporal design of the emotion measurement, it is important to clarify that our objective was not to capture the highly volatile and transient behaviors at a specific crisis peak, but rather to explore the structure of the stabilized cognitive space that the public forms after a public health emergency concludes. Surveying during a relatively stable period effectively reduces the cognitive noise and extreme biases caused by immediate panic. We believe this retrospective consolidation approach provides a more accurate mapping of the panoramic, stabilized cognitive-affective structure. We employed a mixed-methods convenience sampling strategy, distributing the survey both online (via the Wenjuanxing platform) and offline (through in-person distribution by the researchers).

A total of 535 responses were initially collected. To ensure data quality, strict inclusion criteria and quality control procedures were implemented: (1) Cognitive screening questions (e.g., Question 26 regarding nucleic acid testing and Question 35 regarding Fangcang hospitals) were embedded to exclude respondents lacking basic cognitive awareness or attention to the survey; (2) A completion-time threshold was established, and questionnaires completed in less than 2 min were deemed invalid. After rigorous screening, 440 valid questionnaires were obtained, yielding a valid response rate of 82.24%.

Demographically, the sample comprised slightly more females than males. The predominant age group was 18–30 years, aligning well with the primary demographic of active internet users. The majority of respondents held an undergraduate degree or higher and were primarily students or office workers. Regarding internet habits, over half of the participants reported daily internet usage exceeding 6 h, with new media (e.g., TikTok, Zhihu) and social media (e.g., WeChat, Weibo) serving as their primary information channels, highlighting their deep embeddedness in the digital information environment.

Furthermore, reliability and validity analyses were conducted using SPSS 27.0. The questionnaire demonstrated good internal consistency, with Cronbach’s alpha coefficient of 0.838. The Kaiser-Meyer-Olkin (KMO) measure was 0.950 (>0.6), and Bartlett’s test of sphericity was significant (*p* < 0.005). The cumulative variance explained reached 57.892% (>50%), indicating that the instrument possessed excellent content validity and effectively measured the netizens’ cognitive levels in alignment with our research objectives.

## 4 Results

### 4.1 Knowledge dimension analysis

The public’s knowledge reserves play a significant role in resisting online rumors and responding to crises. The knowledge dimension can reflect the public’s level of knowledge mastery in aspects such as prevention and control measures, scientific cognition, and rumor identification, and reveal their cognitive differences, thereby providing a scientific basis for precise intervention.

The overall knowledge mastery of the public is relatively good, with average scores in all dimensions exceeding 3 points. Among them, the detection strategy dimension performed the best. Respondents’ mastery of knowledge with strong daily relevance, such as conventional detection methods and the locations of detection points, was particularly outstanding. However, the scores in the dimension of personal protection and health education were the lowest, especially for the incorrect cognition items such as “drinking white spirit/tea water to fight viruses”, the scores were significantly low. Research shows that respondents have a high level of awareness regarding vaccine types (inactivated /mRNA vaccines), virus variants (Omicron/Delta), and basic prevention and control measures (mask-wearing, health code rules). However, there are significant differences in the understanding of professional terms: the recognition of terms such as “spatio-temporal companion” and “on-site epidemiological investigation” is only at a medium level, and the awareness rate of complex measures such as remote prevention and control and risk area division is also relatively low. At the same time, the awareness levels of management standards for key groups and emergency measures such as silent management and closed-loop operation show a significant stratification phenomenon, reflecting structural differences in the depth of public understanding of the professional prevention and control system.

### 4.2 Thematic dimension analysis

**Figure 9:**
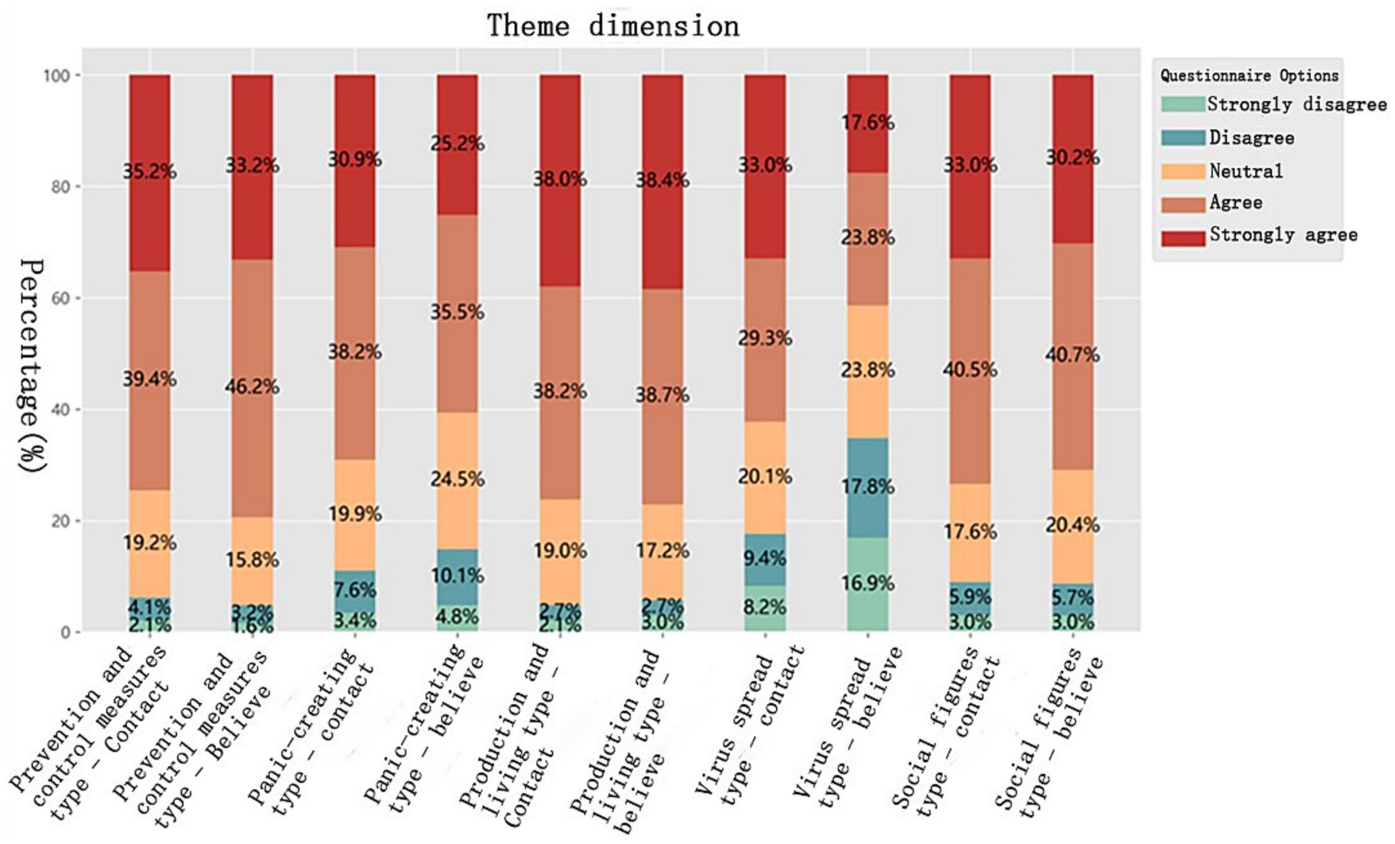
Research results of the topic dimension.

Online rumors usually spread around specific themes, and rumors of different themes have significant differences in their impact on public perception and emotions (Figure 9). The analysis of the thematic dimension helps to reveal the public’s exposure frequency and trust tendency when facing different types of rumors.

The public has come into contact with all five types of rumors, and the proportion of contact is all greater than 50%, which indicates that the contact is relatively high. Among them, the most common type is related to production and life, with a contact rate as high as 76.2%. The possible reason is that such rumors are related to the public’s own work, study and life, so they are the most frequently encountered. At the same time, the belief rate of rumors related to protective measures is the highest, reaching 79.4%. This also proves that when the public is facing the epidemic, they may reduce their thinking due to panic and other reasons, and easily believe some online rumors about protective measures. The belief in rumors about virus spread is the lowest, with less than half of them being 41.4%. This indicates that the public may remain calm and think when facing such rumors due to their own knowledge accumulation or the publicity and refutation by government media, etc., and thus correctly identify the rumors (Figure 9).

### 4.3 Emotional dimension analysis

**Figure 10:**
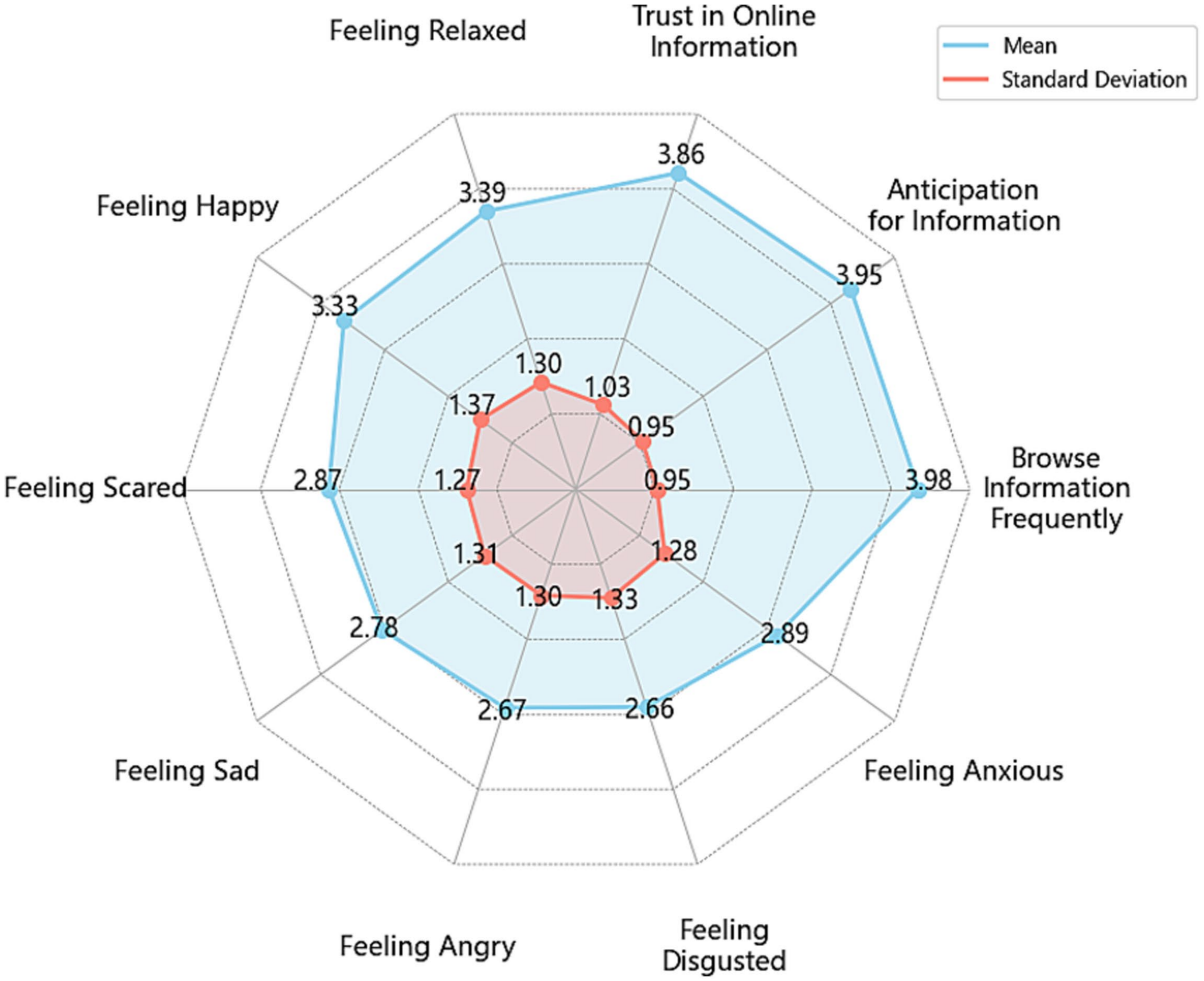
Radar chart of the average score and standard deviation of the emotional dimension.

Public sentiment is a key factor influencing the perception and behavior of individuals toward online rumors. Emotions not only reflect the public’s attitude toward events but also influence their subsequent dissemination and judgment. As shown in the radar chart of average scores and standard deviations for the emotional dimension (Figure 10), the public’s positive emotional scores were generally high during the stabilized period. Specifically, the item “frequently browsing information” received the highest score (*M* = 3.98, SD = 0.95), followed closely by “expecting information updates” (*M* = 3.95, SD = 0.96) and “believing online information” (*M* = 3.86, SD = 1.03). Statistical analysis revealed that the median values for these items were closely aligned with the arithmetic means (e.g., a median of 4.00 for information browsing), suggesting a relatively symmetrical and stable distribution of positive sentiment among the respondents.

These results indicate that a majority of the active netizens maintained a positive outlook and demonstrated a high level of concern for the epidemic’s progress. In contrast, emotional scores for “feeling happy” and “feeling relaxed” were relatively lower, both around 3.3. Scores for negative emotions, such as “fear,” were concentrated between 2.6 and 2.9, with a consistent standard deviation of approximately 1.3. Overall, the public’s emotional tendencies trended from neutral to positive. While certain groups exhibited negative affect, the subgroup demonstrating positive emotions held a slight edge, reflecting a resilient cognitive-emotional structure in the post-pandemic context.

### 4.4 Cognitive space analysis

**Figure 11:**
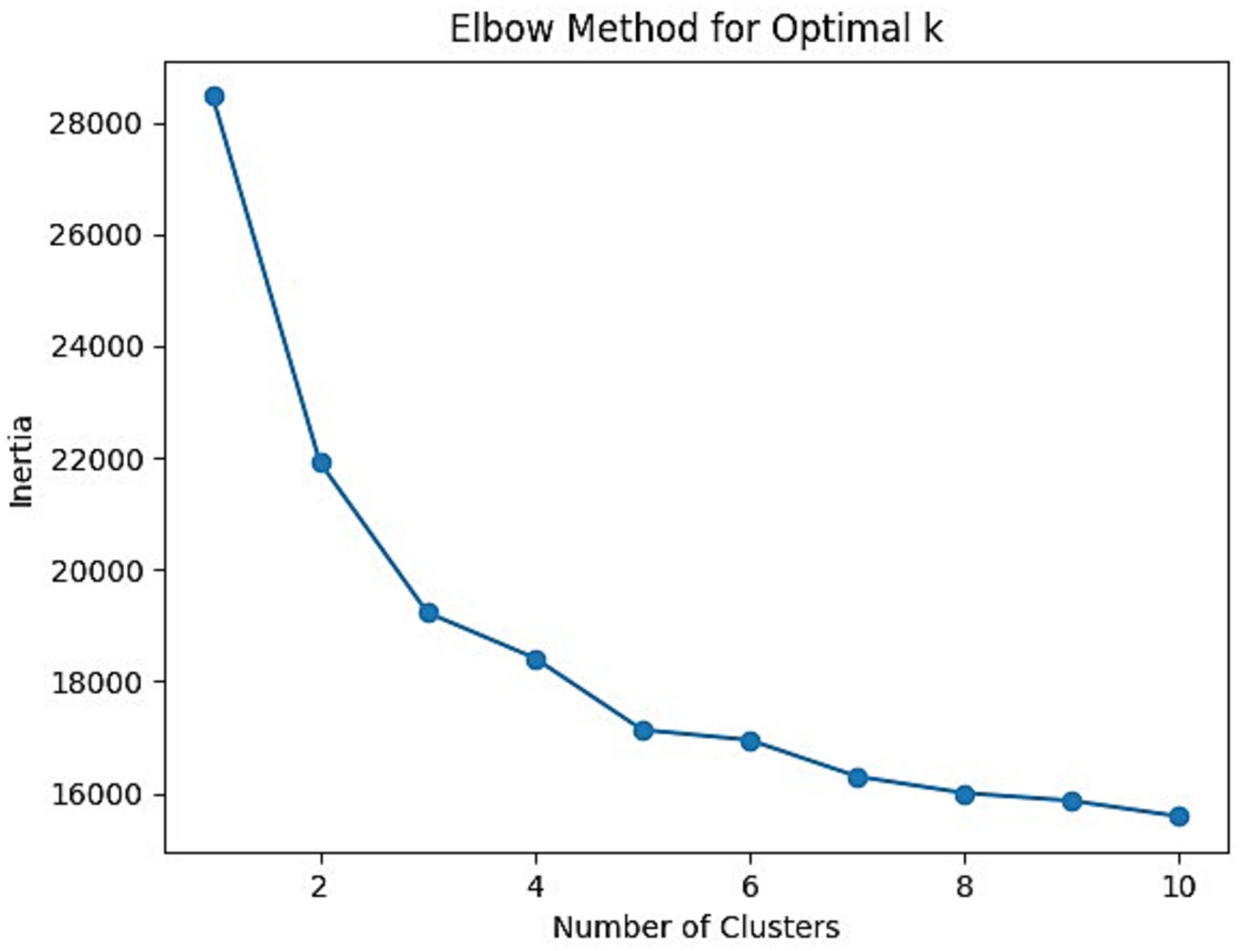
K-means clustering elbow graph.

**Figure 12:**
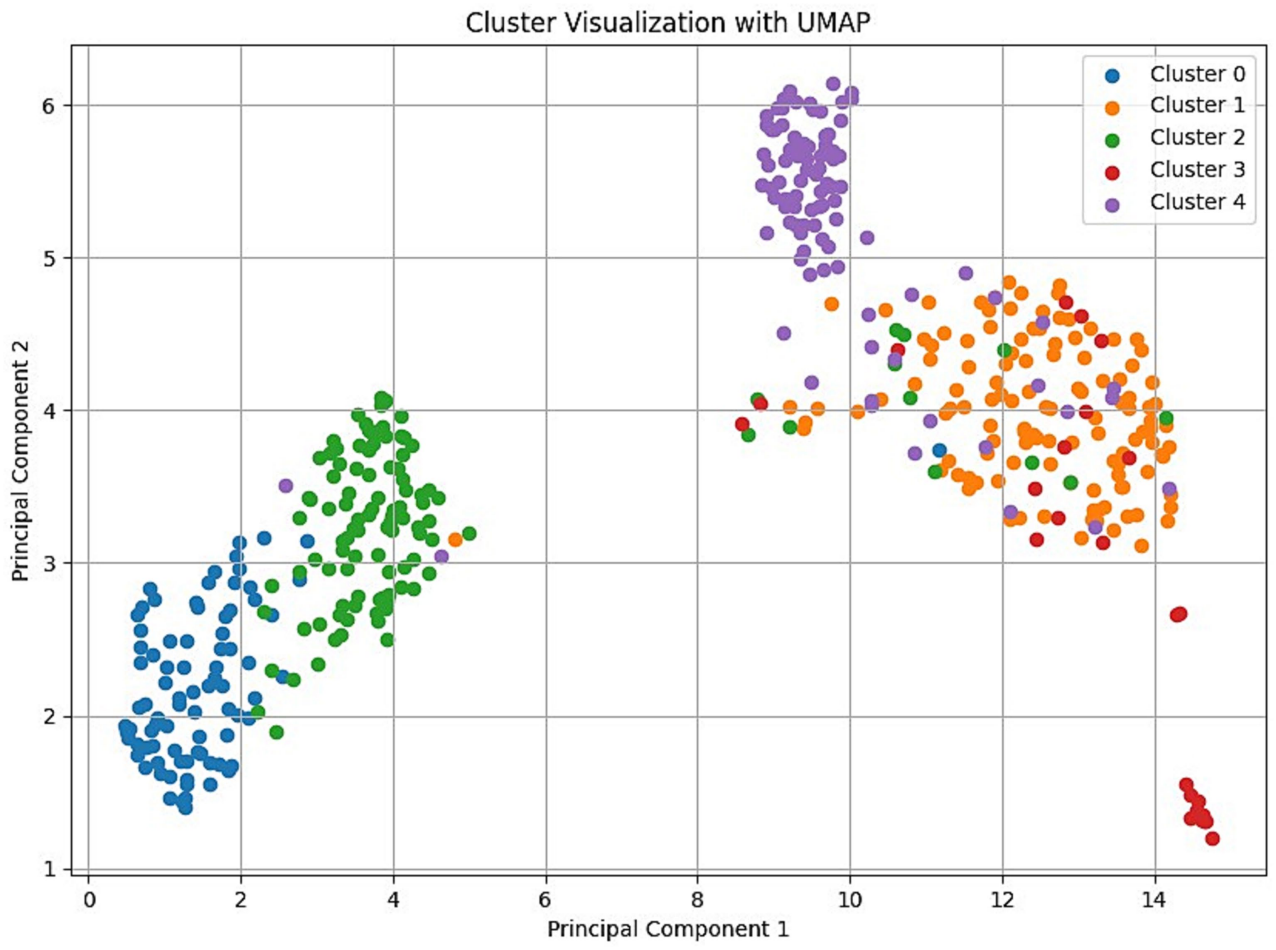
Scatter plot of K-means clusters.

When dealing with online rumors in public health emergencies, an individual’s cognitive level plays a decisive role in behavioral responses. To reveal the intrinsic characteristics of groups with different cognitive levels and identify the roles and functions of various groups in the spread of online rumors, this study selects key indicators in the knowledge dimension, emotion dimension and theme dimension to conduct cluster analysis on the individual cognitive space. When conducting K-means cluster analysis using Python, the elbow graph (Figure 11) visually presents the relationship between the number of clusters and the sum of squared errors (SSE): when the number of clusters varies within the range of 1 to 10, the sum of squared errors continuously decreases with the increase of the number of clusters. Among them, when the number of clusters increases from 1 to 5, the decrease in the curve is significant. When the number of clusters exceeds 5, the downward trend tends to level off. This indicates that when the number of clusters is 5, it can not only ensure the effective distinction of group differences but also avoid the problem of excessively high model complexity caused by excessive division. At this time, the clustering effect is the best. Based on the setting of 5 clusters, the clustering results are presented through a scatter plot (Figure 12), showing the distribution of the data after dimensionality reduction. Different color points represent the five clustering groups from Class 0 to Class 4.

According to the knowledge dimension feature analysis of the clustering results (Table 7), the average scores of Class 0, Class 2, and Class 4 are all above 4, constituting a high knowledge reserve group, and their scores are all at a relatively high level. The scores of Category 1 are all between 3 and 4, belonging to the medium-level group. The average score of Category 3 is lower than 3, belonging to the low knowledge reserve group, with only some scores reaching the average level.

**Table 7:** Knowledge dimension characteristics of clustering categories.

Clustering categories	Etiological and epidemiological characteristics	Vaccination	Personal protection and education	Monitoring and warning	Detection Strategy	Management of infection sources	Prevention and control of key links	Emergency prevention and control measures during the epidemic	Organizational guarantee	Average score
Class 0	4.40	4.37	3.47	3.79	4.39	4.43	4.43	4.38	4.39	4.23
Class 1	3.34	3.41	3.52	3.62	3.86	3.72	3.19	3.59	3.47	3.52
Class 2	4.44	4.35	3.52	4.41	4.42	4.45	4.41	4.43	4.42	4.32
Class 3	2.12	2.46	3.46	2.42	3.29	2.44	2.21	2.32	2.45	2.58
Class 4	4.15	4.17	4.49	4.03	4.28	4.28	4.11	4.43	4.29	4.25

Further distinguishing the differences among the high knowledge reserve groups, the scores of all nine knowledge points in category 4 are above 4, indicating that category 4 has the most complete knowledge reserve. Although the average score of category 2 is slightly higher than that of category 4, it is lower than that of category 4 in “Personal Protection and Education”. Meanwhile, the scores of category 0 in “personal Protection and Education” and “Monitoring and Early Warning” are relatively low, suggesting that the knowledge reserve of category 0 is not comprehensive enough.

According to the topic dimension feature analysis of the clustering results (Table 8), the contact scores of Class 0, Class 2, and Class 4 for each type of topic all reach the level of 4 or above, belonging to the high-contact population. The contact score of Class 1 is at a medium level. However, the contact scores for each theme of Category 3 are all below 3, indicating a low-contact population.

**Table 8:** Clustering category topic dimension features.

Topic dimension indicators	Class 0	Class 1	Class 2	Class 3	Class 4
Prevention and control measures type-contact	4.33	3.62	4.41	2.71	4.19
Prevention and control measures type-believe	4.37	3.78	4.39	2.50	4.25
Panic-creating type-contact	4.29	3.28	4.41	2.11	4.11
Panic-creating type-believe	3.05	3.39	4.38	2.14	4.19
Production and living type-contact	4.36	3.70	4.46	2.43	4.35
Production and living type-believe	4.48	3.78	4.48	2.64	4.03
Virus spread type-contact	4.45	2.77	4.41	2.07	3.90
Virus spread type-believe	1.66	3.00	4.27	2.25	3.28
Social figures type-contact	4.41	3.44	4.43	2.50	4.06
Social figures type-believe	4.41	3.39	4.39	2.32	4.01

Specifically, the high-contact population can be further subdivided. Category 2 shows belief in all types of topics and belongs to the gullible group, while category 4 shows a skeptical attitude in the “virus spread category”, and category 0 shows suspicion in the “panic creation category” but a disbelieving attitude in the “virus spread category”, with a relatively higher degree of rationality. Overall, the degree of contact with Class 1 is slightly lower, so all maintain a skeptical attitude. The degree of contact and trust in Type 3 is both low, showing an indifferent attitude.

According to the emotional dimension feature analysis of the clustering results (Table 9), Class 0 and Class 2 have higher positive scores and lower negative scores, both belonging to the positive population. Both positive and negative emotions of Category 4 are at a relatively high level, indicating that Category 4 belongs to the contradictory group and their emotions are prone to change. Category 1 has both positive and negative emotions at a medium level, belonging to a rational group. They maintain rational analysis, neither blindly optimistic nor overly pessimistic. The positive and negative emotional scores of Category 3 are both at a relatively low level, indicating that they belong to the emotionally alienated group and show an indifferent attitude toward events.

**Table 9:** Affective dimension characteristics of clustering categories.

Clustering categories	Average score of positive emotions	Average score of negative emotions
Class 0	4.39	2.62
Class 1	2.86	2.99
Class 2	4.32	1.63
Class 3	2.04	2.30
Class 4	3.98	4.11

According to the behavioral characteristic analysis of the clustering results (Table 10), the average score of Class 4 is at a relatively high level, and it actively participates in various information behaviors. The average scores of Class 0 and Class 2 are at a medium level. Their scores in the behaviors of verification and active Posting are relatively low, showing the characteristics of passive participation. They only focus on forwarding and do not actively verify and post. Class 1 only shows low propagation attribute characteristics when the verification and attention scores are greater than 3. The scores of all types of behaviors in Category 3 are at a low level, demonstrating the characteristic of indifference.

**Table 10:** Behavioral characteristics of clustering categories.

Behavioral indicators	Class 0	Class 1	Class 2	Class 3	Class 4
I will forward information about the epidemic	4.29	2.67	4.28	1.64	3.95
I will like or comment on the epidemic information	4.40	2.69	4.18	1.75	4.03
I will verify or question the epidemic information	1.58	3.25	1.71	1.82	3.99
I will follow the public accounts, Weibo or Douyin accounts related to epidemic information	4.47	3.20	4.34	2.07	4.01
I will proactively release information or viewpoints related to the epidemic	1.48	2.01	1.64	1.61	3.83
Average score	3.24	2.76	3.23	1.78	3.96

In summary, based on the characteristics of each dimension of the cognitive space regarding public perception of online rumors, this paper categorizes the public into five types: the Active Rational Disseminators, the Neutral Cautious Bystanders, the Highly-Literate Yet Susceptible Amplifiers, the Indifferent and Alienated, and the Contradictory Yet Active Participants. The cognitive space characteristics of these different groups are analyzed and summarized as follows.

The Active Rational Disseminators possess a solid foundation in public health knowledge, being well-versed in practical information such as vaccines and prevention measures. However, they exhibit certain blind spots in preventive knowledge areas like personal protection and surveillance/early warning. This group is widely exposed to various pandemic rumors and can utilize their knowledge advantage to analyze rumors rationally, maintaining a critical stance particularly toward information that incites panic or relates to virus spread. Nonetheless, due to their knowledge gaps, their dissemination behavior lacks verification awareness; they typically rely on authoritative information, forming a “high-trust, low-reflection” dissemination model.

The Neutral Cautious Bystanders have relatively limited knowledge reserves, grasping only some knowledge closely related to personal daily life (e.g., testing strategies). Their exposure to rumors is low, and they show no significant tendency in trust levels, especially toward virus-spread rumors, often adopting an avoidant attitude. Their emotional experience is somewhat blunted, with both positive and negative emotions approaching neutrality. They typically participate in information dissemination through minimal attention and verification behaviors, acting as low-engagement “silent observers.”

The Highly-Literate Yet Susceptible Amplifiers possess relatively comprehensive knowledge, particularly relying on the official prevention and control knowledge system, but pay less attention to individualized knowledge (e.g., personal protection). This “authority-oriented cognition” leads this group to exhibit high exposure and trust toward various rumor themes, even accepting virus-spread rumors uncritically. Due to their singular emotional tendency, strong positive emotions drive them to frequently forward information. However, they often lack verification behaviors, falling into a “knowledge confidence trap” and becoming “amplifiers” of rumor dissemination.

The Indifferent and Alienated group suffers from a severe lack of knowledge, mastering only some most basic protective information. This group shows almost no reaction to pandemic rumors, with their emotional state tending toward freeze, exhibiting “cognitive-emotional double desensitization.” Their information-related behaviors are nearly stagnant, limited to passively receiving fragmented information, forming a self-isolated cognitive space that demonstrates a vicious cycle of “cognitive poverty and social disembedding.”

The Contradictory Yet Active Participants possess a well-rounded knowledge system, with particularly high awareness in areas like personal protection. They can apply professional knowledge to selectively critique information related to virus spread. This group experiences complex emotions, feeling positive due to prevention effectiveness but also harboring persistent worries about potential risks. This contradictory emotional state drives them to frequently forward, verify, and publish information. However, excessive emotional involvement may increase their information processing burden, manifesting as high participation but a susceptibility to information processing difficulties.

Given the differences in the cognitive spaces of these various groups, enhancing the capacity to counter online rumors requires adopting differentiated guidance strategies. For the Active Rational Disseminators, it is necessary to strengthen their knowledge blind spots (e.g., personal protection and surveillance/early warning knowledge) within public health education, precisely push professional information through authoritative channels, and develop embedded rumor verification tools, leveraging their dissemination advantage to construct a two-way intervention mechanism of “knowledge updating—instant correction.”

For the Neutral Cautious Bystanders, strategies should follow the principle of cognitive offloading to design contextualized prevention guidelines, combined with behavioral incentive strategies to build a micro-reward system, using a “low-threshold exposure—gradual involvement” pathway to enhance information participation.

Regarding the Highly-Literate Yet Susceptible Amplifiers, there is a need to develop rumor deconstruction tools that reveal the non-linear relationship between professional knowledge and critical thinking ability, establish a dissemination credit rating system to guide their social influence toward serving scientific information dissemination, and promote a paradigm shift in cognition.

For the Indifferent and Alienated group, community-embedded intervention strategies should be adopted, implanting visualized prevention knowledge in public service scenarios and leveraging familiar social networks to disseminate localized narratives, aiming to repair their state of cognitive desensitization.

For the Contradictory Yet Active Participants, it is essential to integrate algorithm-based filtering technology with community collaboration mechanisms, build personalized information filtering systems to reduce emotional load, incorporate their high-frequency verification behaviors into a distributed rumor-refuting alliance with identity authentication, thereby transforming their excessive participatory energy in a positive direction.

**Figure 13:**
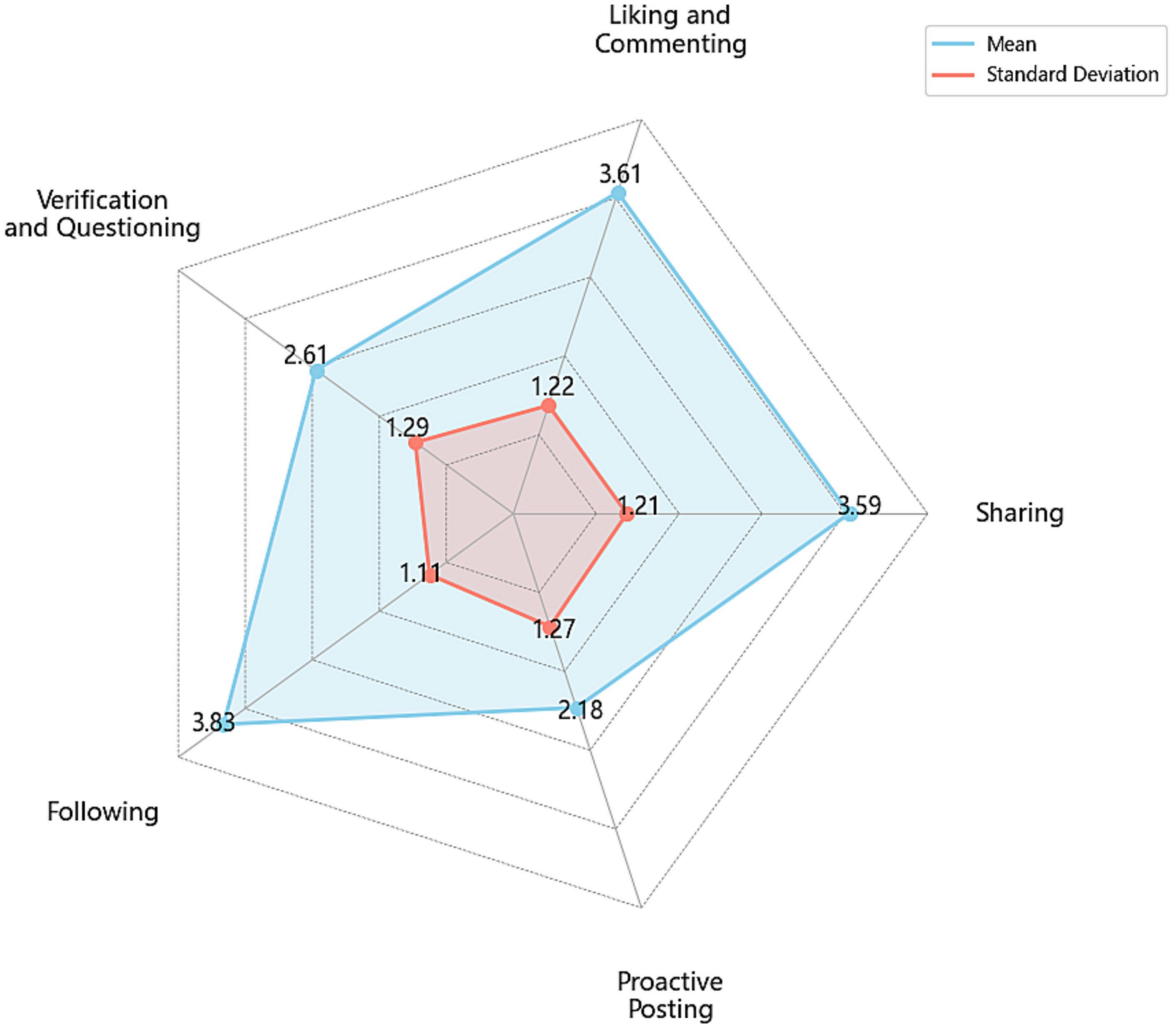
Radar chart of the average score and standard deviation of the behavioral dimension.

Regarding the emotional impact brought by epidemic-related information, the attitudes of participants showed diversity (Figure 13). The average scores of public likes, comments, shares, and follows were all above 3.5, while those of seeking verification, questioning, and active Posting were below 3, with the lowest active Posting as low as 2.18. This can indicate that in the event of the epidemic, Most netizens prefer to participate in information behavior in a passive way and tend to pay attention to information, but they seldom verify the authenticity of the information and do not actively release relevant information.

## 5 Discussion

### 5.1 Main findings

This study, taking online rumors during public health emergencies as its empirical context, is the first to systematically examine the formation mechanisms and evolutionary characteristics of public cognitive structures from the perspective of group cognitive space. Through cluster analysis, the study further reveals the cognitive differences and behavioral patterns among distinct types of online users. The results show that the group cognitive space exhibits pronounced structural, dynamic, and differentiated characteristics, which are reflected in the following three aspects.

First, the group cognitive space demonstrates a three-dimensional structure centered on “knowledge–theme–emotion.” The knowledge dimension determines individuals’ ability to identify and comprehend information (54), the theme dimension reflects the public’s focal concerns and sensitivity to different types of information (55), and the emotional dimension reveals the psychological response mechanisms triggered by information stimuli (56). These three dimensions interact dynamically and collectively construct the overall cognitive framework of netizens during public health events. The findings indicate that, in the early stage of an emergency, the knowledge dimension of the cognitive space exhibits a clear “fragmentation” phenomenon (57). As the event unfolds and official information becomes more transparent, the knowledge structure gradually becomes integrated (58), the thematic focus becomes more concentrated, and emotional volatility weakens, eventually forming a relatively stable cognitive pattern. This suggests that the group cognitive space is not static (59) but is continuously reconstructed under the dual influence of external information stimuli and internal emotional regulation (60).

Based on the proposed cognitive space framework, a cluster analysis of 437 valid samples identifies five distinct groups: rational and active disseminators, cautious and neutral bystanders, highly knowledgeable yet susceptible amplifiers, indifferent and detached individuals, and contradictory yet active participants. These groups differ significantly in terms of knowledge reserve, emotional attitudes, and thematic sensitivity, reflecting the hierarchical differentiation and functional heterogeneity within the group cognitive space. Rational and active disseminators possess higher levels of knowledge and rational judgment but insufficient verification awareness. Highly knowledgeable yet susceptible amplifiers hold strong knowledge reserves but tend to become secondary diffusion nodes due to emotional resonance. Indifferent and detached individuals exhibit the “dual-low” characteristics of low knowledge and weak emotional responses, functioning as “disconnection points” within the information dissemination chain. Contradictory yet active participants show high emotional volatility, actively engaging in information exchange while also being prone to cognitive bias under information overload. These clustering results reveal the internal heterogeneity and interaction patterns embedded in group cognitive space, providing empirical support for understanding the coexistence of rational and irrational behaviors in rumor transmission.

Furthermore, these findings strongly demonstrate the theoretical explanatory value of the “knowledge-theme-emotion” framework. Traditional uni-dimensional studies often fail to explain paradoxical online behaviors, such as why highly educated individuals sometimes participate in rumor spreading. Our framework offers a theoretical explanation for this phenomenon: the cognitive space is governed by the dynamic interplay of its dimensions. For instance, in the “Highly-Literate Yet Susceptible Amplifiers” group, the protective function of the knowledge dimension is effectively bypassed when a specific theme perfectly aligns with a highly arousing emotion. Similarly, the framework explains the behavioral paralysis of the “Indifferent and Alienated” group as a result of cognitive-emotional double desensitization, rather than mere information deficit. Thus, the framework moves beyond descriptive public opinion monitoring, offering a robust theoretical tool to diagnose the root causes of distinct cognitive vulnerabilities and behavioral trajectories in the context of digital infodemics.

The group cognitive space also presents a clear stage-based evolutionary pattern across the lifecycle of a public health event. Results show that, during the outbreak stage, the cognitive space is predominantly driven by negative emotions (61), and public cognition appears highly unstable (62). During the sustained stage, official science communication and accumulated social experience enhance the knowledge dimension and concentrate thematic structures, thereby promoting a process of rational cognitive reconstruction (63). In the declining stage, as event fatigue and economic pressures intensify, the emotional dimension once again becomes the core influencing factor (64) leading to a phenomenon of “re-differentiation” within the cognitive structure. This cyclical pattern of “knowledge accumulation–emotional disturbance–cognitive reconstruction” demonstrates that group cognitive space not only reflects the public’s cognitive pathways for processing risk information but also reveals the dynamic interaction mechanisms among cognition, emotion, and behavior (65). Overall, this study verifies the existence and three-dimensional characteristics of group cognitive space and, through clustering methods, uncovers the internal differentiation and evolutionary logic of distinct cognitive groups (66), thereby offering a new theoretical framework and empirical pathway for advancing research on group cognition.

### 5.2 Implication of the research

The findings of this study contribute to public health crisis communication, online rumor intervention, and social cognition management. First, from the perspective of public health governance, the results suggest that differences in cognitive structures and behavioral responses among various groups constitute an important socio-psychological foundation influencing rumor dissemination (67). Public health authorities should therefore develop stratified and targeted emergency communication strategies based on the structural distinctions within the group cognitive space (68). For example, for rational and active disseminators, cognitive blind-spot supplementation and verification-awareness training can enhance their capacity for rumor identification; for highly knowledgeable yet susceptible amplifiers, reflective communication training based on cognitive feedback mechanisms should be introduced to prevent them from falling into the “knowledge confidence trap”; for indifferent and detached individuals, community-embedded communication, visualized information dissemination, and participation incentives can help reintegrate them into the information exchange network (69). Establishing an intervention chain of “cognitive refinement–behavioral adjustment–trust reconstruction” can effectively improve the public’s risk cognition and information-screening ability during health emergencies.

From the perspective of social cognition and emotional communication, the findings highlight the crucial role of the emotional dimension in the reconstruction of group cognitive space. Negative emotions not only distort individual risk perception but also accelerate rumor diffusion through emotional resonance in social networks. Therefore, public health agencies and media institutions should adopt a dual intervention strategy integrating cognition and emotion. On the one hand, AI-based sentiment detection and online opinion monitoring should be employed to dynamically track emotional fluctuations in digital environments (70); on the other hand, positive emotional guidance and psychological support initiatives should be implemented to mitigate the collective aggregation of fear and anxiety (71). Establishing an emotional feedback mechanism can help stabilize rational cognition and reduce emotion-driven biases in information processing.

From the perspective of social governance system optimization, this study proposes a new framework for rumor intervention based on group cognitive space. Traditional “fact-checking–content refutation” approaches primarily target the information layer, whereas the present research emphasizes multi-level interventions across the cognitive, behavioral, and network layers. At the cognitive level, differentiated health communication strategies should be applied according to group characteristics (72); at the behavioral level, rumor-control mechanisms based on algorithmic recommendations and credibility scoring can be developed; and at the network level, highly participatory groups—such as contradictory yet active participants—should be guided toward positive information dissemination, forming a distributed rumor-refuting alliance and achieving multi-stakeholder collaborative governance. This cognitive-space-oriented intervention logic helps reduce the propagation momentum of rumors at its root. Furthermore, from an ethical perspective, recognizing the distinct cognitive spaces of these groups highlights the severe moral consequences of failing to manage misinformation and disinformation during public health emergencies. Misinformation (false but not intentionally malicious information) and disinformation (deliberately fabricated and misleading information) generate different ethical crises. Failure to manage misinformation violates the ethical principle of beneficence; it allows unintentional rumors to exploit public panic and cognitive blind spots, leading to irrational behaviors, misallocation of scarce medical resources, and delays in critical treatments. Conversely, the unchecked spread of disinformation fundamentally undermines the principles of justice and public autonomy. Deliberate disinformation campaigns manipulate vulnerable cognitive groups (such as the “Highly-Literate Yet Susceptible Amplifiers”), eroding public trust in health authorities and exacerbating social polarization. Therefore, effective rumor intervention is not merely an information management task, but a critical ethical imperative to safeguard social equity and public safety.

Ultimately, this study provides a highly actionable framework for institutions responsible for rumor governance and real-world policy interventions. Moving beyond traditional, one-size-fits-all fact-checking, health authorities and policymakers should translate these descriptive findings into targeted institutional practices based on the “knowledge-theme-emotion” cognitive space. Specifically, institutions must: (1) Pre-crisis phase: Construct customized “knowledge vaccines” databases tailored for highly vulnerable yet unengaged groups (e.g., the Indifferent and Alienated) to preemptively build cognitive immunity; (2) Outbreak phase: Implement real-time thematic diversion strategies to prevent panic-creating topics from monopolizing the cognitive space and paralyzing rational dissemination; and (3) Sustained phase: Transition toward emotional-relational governance by deploying psychological intervention teams alongside traditional rumor-debunking units, particularly to soothe and guide the complex sentiments of “Contradictory Yet Active Participants.” By translating public cognitive structures into these specific policy measures, institutions can shift from reactive rumor suppression to the proactive management of the public cognitive space.

From a theoretical perspective, this study integrates social cognitive theory and dynamic systems theory to propose and empirically validate the concept of group cognitive space, thereby extending the analytical boundaries of information dissemination and socio-psychological interaction in public health emergencies (73). Crucially, the theoretical framework proposed in this article possesses broad cross-domain applicability. Beyond public health crises, it can be widely applied to the governance and handling of various types of public emergencies, such as natural disasters, technological accidents, and major social security incidents, serving as a universal theoretical lens for understanding public cognitive dynamics under high-uncertainty conditions. Furthermore, this framework provides new theoretical support for subsequent interdisciplinary research on cognitive evolution, group emotion, and social behavior, and lays a foundation for future investigations employing neurocognitive analysis, affective computing, and related approaches to uncover the internal mechanisms of cognitive space.

### 5.3 Research limitations

Although this study has made progress in constructing and categorizing the group cognitive space associated with online rumors during public health emergencies, several limitations remain. Regarding the empirical data, our questionnaire study relies on cross-sectional data collected after the pandemic to verify the stabilized cognitive space. While this retrospective snapshot effectively maps the spatial structure, it lacks longitudinal tracking of the same cohort across all dynamic crisis stages. Future research should employ time-series tracking designs to further validate the dynamic evolution of the cognitive space. Second, the empirical sample exhibits certain demographic biases. Because our focus is on online rumor dissemination, the survey targeted “active netizens,” resulting in a sample skewed toward younger, highly educated individuals. Groups with limited internet engagement, such as older adults and children, were not included in this empirical scope. While our proposed theoretical framework possesses broad applicability for general social research, future empirical studies should aim for more diverse and inclusive sampling to enhance the generalizability of the findings across the entire public spectrum. In addition, the rumor dataset was mainly sourced from official rumor-refutation platforms, which may have omitted grassroots rumors that were not documented. This limitation affects data completeness and reduces the ability to fully capture the overall landscape of rumor dissemination.

Prior studies have applied LDA to conduct topic modeling on social media texts and combined it with sentiment analysis to investigate public emotions and topic distributions. However, these studies also noted that such methods struggle to capture deeper psychological motivations and cognitive mechanisms embedded in textual content (10). Similarly, the LDA topic model, BTM algorithm, and other text-mining techniques employed in this study have limitations when dealing with ambiguous semantics, metaphorical expressions, and other complex linguistic features. These constraints may affect the accuracy of topic extraction and sentiment classification.

### 5.4 Future research directions

Based on the findings and limitations of this study, future research can be advanced and expanded in the following directions.

In terms of research subjects and data, future work should broaden sample coverage by including participants of more diverse ages, regions, and educational backgrounds to enhance representativeness. Data sources should also be expanded by incorporating real-time social media data, offline interview materials, and other multimodal information to build cross-scenario datasets and mitigate biases resulting from single-source data.

With respect to research content, further examination of the relationship between cognition and behavior is needed. Future studies may focus on group cognitive processes and cognitive structures, investigate evolutionary patterns and influencing factors across different cognitive stages, clarify how various forms of group cognition shape behavioral decisions, and compare differences in cognitive space across various types of public emergencies.

In terms of research methods and techniques, future studies may integrate Transformer-based text representations and deep learning–based topic models (74) to improve the recognition of ambiguous semantics, metaphorical expressions, and short texts. This would help enhance the precision and robustness of topic identification and sentiment analysis. Additionally, drawing on a quantum social science approach offers a groundbreaking theoretical and methodological direction for future studies. Future research could utilize quantum models (e.g., quantum probability theory) to better capture the complex, superimposed, and non-linear nature of public cognition, behavioral decision-making, and emotional entanglement during high-uncertainty crisis events, thereby providing a more profound explanation for the dynamics of group cognitive space.

## 6 Conclusion

This study provides a new research perspective for understanding online rumors during public health emergencies. While existing studies primarily investigate isolated dimensions—such as theme or emotion—to merely track event trajectories or netizen behavioral characteristics, our study proposes the comprehensive theoretical framework of “Group Cognitive Space.” Specifically, this cognitive space is strongly correlated with the emergency event itself. As the event unfolds and new information emerges, public cognition does not ossify; rather, it continuously reconstructs and evolves. It is only when the event passes and subsides that public cognition gradually solidifies into a stable, describable structure. By examining this stabilized cognitive structure and its characteristics across three core dimensions (knowledge, theme, and emotion), this study elucidates the underlying laws of group cognition formation, thereby providing a robust reference for the handling of public health emergencies. Given current information constraints, this study focuses on these three primary dimensions as a starting point; future research can certainly introduce additional dimensions to further enrich this spatial model.

The research has revealed the dynamic changes in the cognitive space of groups during public health emergencies, but there are still limitations in data collection and processing, such as sample bias and data quality issues. Meanwhile, the existing theoretical framework fails to fully explain the complex changes in public cognition and behavior, and further development is urgently needed. In the future, the relationship between cognition and behavior can be explored from the perspectives of social culture and neuroscience. At the same time, real-time data collection and quantification tools can be optimized to more accurately reflect the dynamic changes in the cognitive space of netizens.

## Data Availability

The raw data supporting the conclusions of this article will be made available by the authors, without undue reservation.
