# Supplementary material for: Group cognitive space of online rumors in public health emergencies: a theoretical and empirical study
Source: Front Public Health. 2026 Jun 12;14:1753674. doi: 10.3389/fpubh.2026.1753674 (PMC13303009; doi:10.3389/fpubh.2026.1753674)
Supplement: Supplementary file 1 [file Data_Sheet_1.PDF]

Public Perception Survey on Public Health Emergencies

Dear Respondent,

Hello! We are a research team from Nanjing University of Posts and Telecommunications conducting an academic study on public perception and cognitive responses during public health emergencies. Thank you very much for your willingness to participate in this survey titled **"Public Perception Survey on Public Health Emergencies."**

This survey aims to better understand how individuals perceive, interpret, and emotionally respond to information related to public health emergencies. Your responses will contribute to advancing research on public cognition, risk communication, and public mental health resilience in crisis contexts.

The questionnaire is anonymous, and all data will be used strictly for academic research purposes. There are no right or wrong answers—please respond based on your genuine experiences and perceptions.

We sincerely appreciate your time and valuable contribution to this research.

A. Your Basic Information

Please check the corresponding box based on your actual circumstances.

A1、**Your Gender [Single Choice]***

○Male

○Female

A2、**Your age [Single-choice question]***

○Under 18 years old

○18-40 years old

○41-60 years old

○Over 61 years old

A3、**Your education level [Single-choice question]***

○Primary school or below

○Junior high/High school

○Technical secondary school/Vocational college/College

○Bachelor's degree/Graduate student or above

A4、**Your current occupation [Single-choice question]***

If retired, please fill in your occupation before retirement.

○Student

○Corporate employee

○Self-employed

○Government or public institution

○Freelancer

○Other ____________________ *

B-**Your Internet Usage Habits**

B1、**Your main channel for obtaining information [Single-choice question]***

○New media (TikTok, Kuaishou, Zhihu)

○Online media (news websites, forums)

○Social media (QQ, WeChat, Weibo)

○Traditional media (TV, newspapers, radio, phone, SMS)

B2、**The mobile internet application (APP) you use most often [Single-choice question]** *

○Entertainment (TikTok, Kuaishou, Xiaohongshu)

○News (Toutiao, Phoenix)

○Social (WeChat, Weibo, QQ)

○Games (King of Glory, Peace Elite)

○Lifestyle (Taobao, Alipay)

B3、**How much time do you spend online each day [Single Choice]***

○ Less than 2 hours

○ 2-6 hours

○ 6-9 hours

○ More than 9 hours

B4、**Have you come across any online rumors related to the pandemic [Single Choice]***

○Yes

○No

B5、**Which information sources do you find most credible? [Single Choice]***

○ New Media (Douyin, Kuaishou, Xiaohongshu)

○ Online Media (News Websites, Forums)

○ Social Media (QQ, WeChat, Weibo)

○ Traditional Media (Television, Newspapers, Radio, Phone, SMS)

B6、**Are you willing to participate in discussions related to the pandemic? [Single Choice]***

○ Very unwilling

○ Unwilling

○ Neutral

○ Willing

○ Very willing

C. Public Perception Survey

C: Please assess how closely the following statements align with your actual situation based on your feelings, and mark "√" in the corresponding option.

1. Your emotional state over the past week

-----1-----	-----2-----	-----3-----	-----4-----	-----5-----
**Rarely**	**Occasionally**	**Sometimes**	**Often**	**Almost Always**

**Emotional States**	**1**	**2**	**3**	**4**	**5**
During the past week, I felt calm and relaxed.	1	2	3	4	5
During the past week, I felt joyful and able to enjoy things.	1	2	3	4	5
During the past week, I felt happy and content.	1	2	3	4	5
During the past week, I felt angry or irritated.	1	2	3	4	5
During the past week, I felt sad or distressed.	1	2	3	4	5
During the past week, I felt fearful or anxious.	1	2	3	4	5

2. Your understanding of COVID-19-related knowledge

-----1-----	-----2-----	-----3-----	-----4-----	-----5-----
**Strongly Disagree**	**Disagree**	**Neutral**	**Agree**	**Strongly Agree**

(1) Etiological and Epidemiological Characteristics

Questionnaire Items	1	2	3	4	5
I am familiar with emerging SARS-CoV-2 variants such as Omicron, Delta, and XBB.	1	2	3	4	5
I understand that COVID-19 can involve asymptomatic infection.	1	2	3	4	5
I understand that COVID-19 can be transmitted through airborne or aerosol routes.	1	2	3	4	5
I understand that COVID-19 infection may lead to complications such as organ failure or white lung syndrome.	1	2	3	4	5

(2) Vaccination

Questionnaire Items	1	2	3	4	5
I am familiar with different types of COVID-19 vaccines, including inactivated vaccines (e.g., Sinovac, Sinopharm), mRNA vaccines, and adenovirus vector vaccines (e.g., CanSino).	1	2	3	4	5
I am aware of recommended precautions prior to vaccination (e.g., avoiding alcohol consumption or certain dietary practices).	1	2	3	4	5
I understand that vaccination may be associated with potential side effects such as headache, hair loss, or decreased appetite.	1	2	3	4	5

(3) Knowledge of Personal Protection and Health Education

Questionnaire Items	1	2	3	4	5
I understand pandemic prevention and control measures, such as wearing masks in public and maintaining social distancing.	1	2	3	4	5
I understand that frequent consumption of strong liquor (e.g., Baijiu) or tea can effectively protect against the COVID-19 virus.	1	2	3	4	5
I understand the latest pandemic prevention and control measures.	1	2	3	4	5

(4) Monitoring and Early Warning

Questionnaire Items	1	2	3	4	5
I understand the coding rules for red and yellow Health Codes.	1	2	3	4	5
I know the body temperature threshold for COVID-19 screening and early warning.	1	2	3	4	5
I am familiar with terms such as "spatiotemporal companion" and "on-site epidemiological investigation".	1	2	3	4	5

*(**"spatiotemporal companion":** Individuals whose phone signals overlapped with those of confirmed cases in time and space)*

(5) Testing Strategy

Questionnaire Items	1	2	3	4	5
I understand routine testing procedures such as antigen testing and nucleic acid testing.	1	2	3	4	5
I am familiar with the pandemic testing sites in my area.	1	2	3	4	5
I understand how to use testing kits.	1	2	3	4	5

(6) Management of Infection Sources

Questionnaire Items	1	2	3	4	5
I understand centralized quarantine and home quarantine.	1	2	3	4	5
I understand the situation of confirmed cases in my area.	1	2	3	4	5
I understand the criteria for determining close contacts.	1	2	3	4	5

(7) Prevention and Control in Key Settings

Questionnaire Items	1	2	3	4	5
I understand the classification of key populations, such as medical personnel and students, in pandemic prevention and control.	1	2	3	4	5
I understand the classification criteria for risk areas.	1	2	3	4	5
I understand remote prevention and control measures.	1	2	3	4	5

(8) Emergency Prevention and Control Measures During an Outbreak

Questionnaire Items	1	2	3	4	5
I understand Fangcang shelter hospitals.	1	2	3	4	5
I understand the measures for virus disinfection.	1	2	3	4	5
I understand emergency pandemic prevention and control measures such as static management and closed-loop management.	1	2	3	4	5

*(**Fangcang shelter hospitals**: Temporary large-scale healthcare facilities used for isolating and treating patients with mild to moderate symptoms.)*

*("**Static management**" refers to strict movement restrictions or lockdowns, while "closed-loop management" restricts individuals to specific operational bubbles to prevent virus transmission.)*

(9) Organizational Support

Questionnaire Items	1	2	3	4	5
I understand the official policies and notices regarding pandemic prevention and control.	1	2	3	4	5
I understand the various levels of organizations involved in pandemic prevention and control.	1	2	3	4	5
I understand the recruitment of pandemic control volunteers.	1	2	3	4	5

3. Emotional and Behavioral Characteristics Triggered by Epidemic Information

-----1-----	-----2-----	-----3-----	-----4-----	-----5-----
**Strongly Disagree**	**Disagree**	**Neutral**	**Agree**	**Strongly Agree**

(1) Emotional Characteristics

Questionnaire Items	1	2	3	4	5
I frequently browse pandemic-related information.	1	2	3	4	5
I look forward to updates on pandemic-related information.	1	2	3	4	5
I trust the pandemic information I encounter online.	1	2	3	4	5
Pandemic-related information makes me feel **relaxed**.	1	2	3	4	5
Pandemic-related information makes me feel **happy**.	1	2	3	4	5
Pandemic-related information makes me feel **fearful**.	1	2	3	4	5
Pandemic-related information makes me feel **sad**.	1	2	3	4	5

Questionnaire Items	1	2	3	4	5
Pandemic-related information makes me feel **angry**.	1	2	3	4	5
Pandemic-related information makes me feel **disgusted**.	1	2	3	4	5
Pandemic-related information makes me feel **anxious**.	1	2	3	4	5

(2) Behavioral Characteristics

Questionnaire Items	1	2	3	4	5
I forward or share pandemic-related information.	1	2	3	4	5
I like or comment on pandemic-related information.	1	2	3	4	5
I verify or question pandemic-related information.	1	2	3	4	5
I follow social media accounts (e.g., WeChat official accounts, Weibo, or Douyin/TikTok) for pandemic-related information.	1	2	3	4	5
I actively post pandemic-related information or my own opinions.	1	2	3	4	5

4. Finally, you will see five pieces of information. Please read the texts carefully and answer the related questions (Thematic Dimension)

-----1-----	-----2-----	-----3-----	-----4-----	-----5-----
**Strongly Disagree**	**Disagree**	**Neutral**	**Agree**	**Strongly Agree**

(Appendix notes: Academician Zhong Nanshan and Dr. Zhang Wenhong are prominent and highly trusted public health figures and infectious disease experts in China.)

(1) In early 2020, following the outbreak of COVID-19, Bai Yansong hosted a live special broadcast on "Novel Coronavirus Pneumonia" on the CCTV News Channel, inviting Academician Zhong Nanshan to introduce the current status of pandemic prevention and control and the upcoming tasks.

Questionnaire Items	1	2	3	4	5
I have been exposed to this type of information.	1	2	3	4	5
I believe the content of this type of information.	1	2	3	4	5

(2) In June 2020, a cluster outbreak occurred in Xinfadi, Beijing. A total of 26,350 close contacts were traced, and all 562 related contacts in Beijing were placed under centralized quarantine and medical observation.

Questionnaire Items	1	2	3	4	5
I have been exposed to this type of information.	1	2	3	4	5

Questionnaire Items	1	2	3	4	5
I believe the content of this type of information.	1	2	3	4	5

(3) In early 2021, the Nanjing Municipal Party Committee and Municipal Government issued an emergency notice declaring Nanjing to be in a "wartime" state. Personnel returning to Nanjing were required to provide a negative nucleic acid test certificate obtained within 7 days, in addition to completing a 21-day medical observation period.

Questionnaire Items	1	2	3	4	5
I have been exposed to this type of information.	1	2	3	4	5
I believe the content of this type of information.	1	2	3	4	5

(4) In December 2021, Zhang Wenhong pointed out that to minimize the inhalation of ethylene oxide, the correct way to wear a mask is to tear open the packaging bag, shake the mask in the air, and let the ethylene oxide volatilize as much as possible to reduce carcinogenic residues.

Questionnaire Items	1	2	3	4	5
I have been exposed to this type of information.	1	2	3	4	5
I believe the content of this type of information.	1	2	3	4	5

(5) In September 2022, express delivery services were suspended in several districts and counties of Chengdu (e.g., Qingyang, Jinniu, Chenghua, Xindu, Pujiang, Wuhou, Jinjiang, and Longquanyi). Due to the presence of the COVID-19 virus on the outer surfaces of express packages, multiple couriers tested positive via antigen tests, and most express delivery outlets were temporarily suspended according to pandemic prevention and control requirements.

Questionnaire Items	1	2	3	4	5
I have been exposed to this type of information.	1	2	3	4	5
I believe the content of this type of information.	1	2	3	4	5

Special Reminder:

Items in this section include a mix of factual official news (e.g., Items 1 and 2) and widespread pandemic-related misinformation (e.g., Item 4 regarding mask carcinogens) to assess respondents' information discernment capabilities.

If you encounter the spread of related information, please debunk it in time to avoid spreading!
